# An Alum-Free Jellyfish Treatment for Food Applications

**DOI:** 10.3389/fnut.2021.718798

**Published:** 2021-08-23

**Authors:** Gianluca Bleve, Francesca Anna Ramires, Stefania De Domenico, Antonella Leone

**Affiliations:** ^1^Unità Operativa di Lecce, Consiglio Nazionale delle Ricerche-Istituto di Scienze delle Produzioni Alimentari, Lecce, Italy; ^2^Department of Biological and Environmental Sciences and Technologies (DiSTeBA), University of Salento, Lecce, Italy; ^3^Consorzio Nazionale Interuniversitario per le Scienze del Mare (CoNISMa), Local Unit of Lecce, Lecce, Italy

**Keywords:** edible jellyfish, novel food, safety assessment, quality traits, nutritional traits, novel food regulation

## Abstract

Jellyfish, marketed and consumed as food in The Far East, are traditionally processed using salt and alum mixtures. In recent years, the interest of Western consumers in jellyfish (JF) as a food source is increasing. In Europe [European Union (EU)], JF-derived food products are regulated by a novel food law, but methods for JF treatment and processing have not been developed yet. In this study, a protocol for the stabilization and processing of JF into semi-finished food products without the use of alum is proposed for the first time. Safety and quality parameters, together with a series of technological and nutritional traits, were used to monitor the proposed process and for the characterization of the JF-derived products. Calcium lactate (E327), calcium citrate (E333), and calcium acetate (E263), which are food thickening/stabilizing agents allowed by EU regulations, were used in order to control the presence of possible microbial pathogens and spoilage species. The use of calcium lactate and citrate led to an increase in texture values (~1.7–1.8-fold higher than in starting raw materials) and in several nutritional traits such as antioxidant activity, and protein and fatty acid content. In particular, the combination of JF treatments with calcium salts and phenolic compounds resulted in an antioxidant activity increase of up to 8-fold, protein concentration increase of up to 2.6-fold, fatty acid composition maintenance, and a ω6/ω3 ratio lower than 1. For the first time, the application of phenolic compounds to improve JF technological and nutritional features was verified. This study proposes a new procedure for JF treatment and stabilization useful for future potential food applications in Western countries.

## Introduction

The presence of jellyfish (JF) in various marine environments seems to be increasing, mainly in response to the ongoing climate change and reduction in the number of predators. The abnormal increase in JF populations, named “blooms,” can be problematic for fishing operations, aquaculture plants, and other anthropic activities in coastal areas, mainly for small-scale fishery (1).

JF are considered a traditional food in Asian countries, such as China, Japan, Korea, and Thailand, which host most JF consumers and producers (2). Although several studies on these preparations have already been reported, the processing methods and techniques, which are part of a long and often undisclosed tradition, can vary according to species, producers, and market requirements (3–7).

In order to prepare JF for human food consumption, freshly caught JF need to be processed within hours, since they have a high susceptibility to spoilage. Oral arms are separated by the umbrellas and washed with a high volume of water to remove mucus, gonads, sand, and bacteria. Then, they are traditionally soaked in a mixture of NaCl and aluminum salts [KAl(SO_4_${)}_{2}^{*}$12H_2_O or AlNH_4_ (SO_4_${)}_{2}^{*}$12H_2_O], in a ratio that varies depending on the methods used (2, 8, 9). This operation is repeated several times, thus gradually reducing the proportion of alum salts. As a consequence, this procedure reduces the water content and changes the JF gelatinous tissue into the consistency expected for the final edible product, generally characterized by a crispy and firm texture highly appreciated by the Eastern market. Alum metal ions can act both by modifying JF tissue mechanical and chemical properties, producing rubber-like hardening effects, probably by cross-linking collagen JF fibers, and partly disinfecting JF materials (8). Depending on the JF species and the adopted treatment, process duration can last from 4 to 40 days. Nowadays, JF food production is still considered as an empirical method or even as an art passed down from master custodians of ancient recipes (4, 10).

In some cases (such as in some food companies in the United States), automation has been performed to reduce process time, and different organoleptic and sensorial (color, taste, texture) traits have been developed in response to buyers and emerging markets. Before consumption, salt-preserved and semi-dried JF products are partially rehydrated by soaking in water several times. These products are generally served in salads and soups, together with sauces (sesame oil, soy sauce, vinegar, etc.), since they have little or no flavor. Recently, ready-to-eat or ready-to-use JF products are marketed to be consumed without soaking, accompanied by sauces (2).

In 2015, the United States Department of Agriculture (USDA) considered edible JF similar to vegetables, such as broccoli and carrots, and included them as a natural diet food. Rehydrated JF show content of 92–96% water, 3–7% protein, and negligible carbohydrate, fat, and cholesterol levels (2). Calcium, magnesium, potassium, and sodium are the most present macro-elements, but once processed JF food products also contain elevated aluminum levels due to the alum curing agent (11, 12). The long exposure of JF tissues to the curing agent, during processing, shipping, and storage, increases the salt penetration and tissue-binding of aluminum ions, resulting in a very high content of this metal. In China, a study revealed that JF in the ready-to-eat form contained very high aluminum levels, from 400 to 1800 mg/kg with a mean of 1,200 mg/kg, easily exceeding the provisional tolerable weekly intake (PTWI) (13), whereas a recent study has shown an aluminum content between 75 and 124 mg/kg of edible JF (14).

Epidemiological investigations revealed that aluminum can cause memory impairment and cognitive dysfunctions, which could lead to neurodegenerative diseases such as Alzheimer's and Parkinson's disease (15–19). Furthermore, a reduction in dietary intake of aluminum is highly recommended, since it can provoke high levels of aluminum in plasma that can enter the central nervous system through the blood-brain barrier and accumulate in the brain (20). In Europe, a tolerable weekly intake (TWI) was established at 1 mg aluminum/kg body weight/week; however, the limits could even be more restrictive for overexposed populations (17, 19, 21). According to these limits, all JF-based products currently in the market would probably exceed the aluminum levels authorized in Europe. Additionally, dietary aluminum intake should be considered in the decision-making process for the admission of novel alum-treated foods for the European population.

In Europe, like in many other Western countries, JF are not a traditional food, and they are not usually consumed. The JF market is probably limited to Asian communities. In the last years, western food markets and some western population with a long and consolidated culinary tradition, such as Italians, have shown interest in JF as food (22, 23). The production of ingredients from the *Catostylus tagi* JF for the preparation of a new ready-to heat snack was also proposed (24). However, the use and sale of JF in Europe are still hindered by the regulation on novel food (25) and by the absence of standard methods for the treatment and processing of the raw material according to EU safety standards. In the previous study conducted by the authors, a set of parameters suitable for the risk assessment of JF as food in Europe was proposed (26), since European regulation on seafood safety did not mention JF and JF-derived products among permitted foodstuffs (27, 28).

Among several possible firming, thickening, and stabilizing agents, the use of calcium salts to treat JF tissues, mainly composed of collagen, is expected to reduce enzymatic activities and the development of undesired microorganisms as well as improve the texture and nutraceutical traits of the semi-finished product. It was demonstrated that both mechanical and structural properties of the collagen fibril show prominent dependence on concentrations of calcium ions. In particular, Ca^2+^ ions can tightly couple with the collagen fibril by chelation with its negatively charged groups (carbonyl and carboxyl groups) to form chelate rings (29).

In food matrices, polyphenols are known to form complexes with proteins, leading to changes in the structural, functional, and nutritional properties of both compounds (30). They can interact with proteins *in vitro* and *in vivo*, affecting their antigenicity, digestion, enzymatic activity, structure, and quality (31, 32). Many *in vitro* studies have shown that these interactions are mainly non-covalent hydrophobic and may be subsequently stabilized by hydrogen bonding (33–35). However, the interaction between proteins and plant phenols can also lead to covalent bonds because of the capability of phenols to form quinone or semi-quinone radicals. The reaction proceeds with polymerization (36). In a study, the stabilization of type I collagen was investigated using the plant polyphenol catechin: shrinkage at 70°C demonstrated that catechin was able to impart thermal stability to collagen (37).

This study, for the first time, develops a procedure to process JF raw material without using alum as a firming and stabilizing agent in order to obtain semi-finished products suitable for subsequent food applications. Based on previous studies aimed at obtaining food ingredients for human consumption (26, 38–40), the edible sea lung or barrel JF *Rhizostoma pulmo* was chosen as model organism. Calcium salts, selected from the food additive list permitted in EU, USA, Australia, and New Zealand, were tested as firming and stabilizing agents for JF biomasses.

In this study, the use of phenolic compounds such as rutin and ferulic acid was proposed as additional tissue stabilizing agents after calcium salt treatment. This further procedure also showed an improvement in the nutraceutical features of the product. The obtained products were characterized at the microbiological, technological, and nutritional levels. Here, for the first time, an effective processing strategy for obtaining JF semi-finished food products, in accordance with safety and quality requirements of the current EU regulations, is presented.

## Materials and Methods

### Sample Collection and Pre-treatment

*Rhizostoma pulmo* JF specimens were collected from an open boat on the coast of Ionian Sea, at Marina di Ginosa (Taranto, Italy) (40°24'36.8“N 16°53'04.0”E) with a 3-5 cm mesh nylon fishing net, during samplings in 2018 and 2019 summer period. Alive JF were temporarily stored at 10–15°C immersed in seawater in food-grade containers and transported to the laboratory within 3 h after collection. In the laboratory, the JF were immersed in sterile seawater (SW), and the umbrella and oral arms were separated with a plastic knife to remove the content of the digestive cavity. The umbrella and oral arms were washed three times with sterile seawater (JF washed with sterile sea water, JFSW) and then submitted to treatment in newly formulated brines with firming agents or stored in sterile plastic bags at −80°C.

### Microbiological Analyses

Microbiological analyses on JF tissue were performed following the procedure already described by Bleve et al. (26), with some modifications. The samples were subjected to serial dilutions with 1 g/L (w/v) peptone water. The diluted samples were applied on agar slants containing the following media: plate count agar (PCA, Lab M, Lancashire, United Kingdom) for total bacterial count added with 0.05 g/L nystatin (Sigma-Aldrich, Darmstadt, Germany) and incubated at 30°C for 48–72 h; violet red bile glucose agar (VRBGA, Lab M, Lancashire, United Kingdom) for Enterobacteriaceae identification by incubation at 37°C for 18–24 h; violet red bile agar (VRBA, Lab M, Lancashire, United Kingdom) for the detection and enumeration of coli–aerogenes bacteria incubated at 37°C for 24–48 h; Baird Parker Agar Base (BP, Lab M, Lancashire, United Kingdom) for the enumeration of coagulase-positive staphylococci incubated at 37°C for 24–48 h; mannitol salt agar (MSA, Lab M, Lancashire, United Kingdom) for the isolation of pathogenic staphylococci incubated at 37°C for 18–72 h; thiosulfate citrate bile sucrose agar (TCBSA, Sigma-Aldrich, Darmstadt, Germany) for the detection and enumeration of *Vibrio* spp. incubated at 37°C for 18–24 h; Bacillus ChromoSelect Agar (BCSA, Sigma-Aldrich, Darmstadt, Germany) added with polymyxin B supplement for the enumeration of *Bacillus* spp. incubated at 30°C for 24–48 h; iron agar (Lyngby) without cysteine (Sigma-Aldrich, Darmstadt, Germany) for the detection and enumeration of hydrogen sulfide-producing bacteria incubated at 25°C for 48 h; *Pseudomonas* agar (Lab M, Lancashire, United Kingdom) added with CFC supplement (Lab M, Lancashire, United Kingdom) for the enumeration of *Pseudomonas* spp. incubated at 35°C for 24–48 h. The enumeration of yeast and molds were performed by incubation at 25°C for 5 days on Dichloran Rose-Bengal Chloramphenicol Agar (DRBC, Thermo Fisher Scientific, Monza, Italy).

For the determination of halophilic microorganisms, the procedure described by Bleve et al. (26) was followed. Briefly, all the samples and their respective serial dilutions were plated in R2A (Sigma-Aldrich, Darmstadt, Germany), marine agar (peptone 5 g/L, yeast extract 1 g/L, agar 16 g/L) added with 0.05 g/L of nystatin and incubated at 30°C for 48–72 h for bacteria, or added with 0.1 g/L of ampicillin and 0.05 g/L of kanamycin and incubated at 25°C for 2–7 days for fungi; sabouraud dextrose agar (SDA, Lab M, Lancashire, United Kingdom) and corn meal agar (CMA, Sigma-Aldrich, Darmstadt, Germany) added with 0.1 g/L of ampicillin (Merck KGaA, Darmstadt, Germany) and 0.05 g/L of kanamycin (Merck KGaA, Darmstadt, Germany) and incubated at 25°C for 2–7 days. Artificial seawater (3% NaCl, 0.07% KCl, 1.08% MgCl_2_, 0.54% MgSO_4_, 0.1% CaCl_2_, w/v) (41) was added to the CMA and SDA media. For each plate, the number of colony-forming units (CFUs) per gram of JF was determined.

### Molecular Identification of Bacterial Isolates

The bacterial total genomic deoxyribonucleic acid was extracted using the Power Soil DNA Isolation Kit (MO BIO, Carlsbad, CA, United States) following the protocol of the manufacturer. The 16S rDNA region was amplified according to Bleve et al. (42). The amplicons were purified, and DNA sequencing was performed as previously described by Bleve et al. (43). The sequences were analyzed with Chromas program version 1.45 (www.technelysium.com.au) and the BLAST program (https://blast.ncbi.nlm.nih.gov/Blast.cgi?PROGRAM=blastn&PAGE_TYPE=BlastSearch&LINK_LOC=blasthome) for sequence alignment, and compared with the sequences in the GenBank database (Release 233).

### JF Treatments

Brine treatments. The brines were formulated using solutions of an alpha-hydroxy organic acid calcium salt. Three different types of brines were formulated as described below, calcium citrate, calcium lactate, and calcium acetate, all from Sigma-Aldrich (Darmstadt, Germany).

The solutions of calcium citrate, lactate, and acetate were formulated using 0.1 M calcium citrate solution, 0.1 M calcium lactate hydrate solution, and 0.1 M calcium acetate solution, and the pH was adjusted to 5 using the corresponding alpha organic acid 1 M citric acid, 1 M lactic acid 85% (v/v), and 0.1 M glacial acetic acid solutions, all from Sigma-Aldrich (Darmstadt, Germany). The JF (umbrella and oral arms), immediately after sampling, were extensively washed with sterile seawater (hereafter named JFSW) and used for brine treatments. In order to obtain homogeneous and comparable samples, JF specimens were dissected as described in Leone et al. (39). Briefly, each JF specimen was separated into quarters, and each quarter was subjected to different brine treatments. At least five JF specimens (about 30 cm in diameter), separated into quarters, were used in each batch for each independent experiment. Combined batches of umbrellas and oral arms of fresh JFSW were immersed in brines in a 1:1 ratio (v/v) JF tissue: brine in food-grade glass or plastic containers. During the experiment, the containers were maintained in a refrigerated chamber, at 4°C; the analyses and tests were performed at 0, 1, 5, and 10 days of treatment. At each point of the time course (days 0, 1, 5, and 10), a representative part of each of the JF samples (both umbrella and oral arms) was removed from the container and analyzed for texture, pH, salinity, antioxidant activity, protein and lipid content, fatty acid composition, and microbiological counts. At the end of the experiment, the remaining samples were sealed in food-grade plastic bags and stored at −80°C for further analyses. Additionally, in order to verify the effect of −80°C freezing, parallel batches of JFSW samples were stored at −80°C and, after thawing on ice (0°C, overnight), treated with brines in the same way as described above.

Brine and phenol treatments. A set of JF samples was processed for 10 days in brines with the procedure described above and further treated with phenol compounds, specifically, ferulic acid (Sigma-Aldrich, Darmstadt, Germany) or rutin hydrate (Sigma-Aldrich, Darmstadt, Germany). Solutions containing each phenolic compound were prepared at the final concentration of 2 mg/g (0.2% w/w of FW) of treated JF (44) in sterile distilled water (pH 7) by considering a theoretical collagen content of about 5–10% (w/v) in JF tissues. In summary, each JF sample was treated for 10 days with the different calcium salts before being washed with sterile water; successively, their volume was approximately measured and a solution of either ferulic acid or rutin was added in a ratio 0.25:1 (v/v) to the JF sample. The JF tissues in phenol solutions were incubated at 4°C for 24 h. At the end of incubation, the JF tissues were drained off and analyzed for texture, pH, salinity, antioxidant activity, protein and lipid content, fatty acids composition, and microbiological load.

### Physicochemical Analysis

Salinity, pH, and texture were evaluated at days 0, 1, 5, and 10 of brine treatment, and at 10 days + 1 day of incubation in the samples treated with either a ferulic acid- or rutin-containing solution.

Salinity was measured by using a salinity refractometer for seawater and marine aquaria 0–10% hydrometer with automatic temperature compensation RHS-MR110 ATC (45). The texture was measured with a digital texturometer (model 53205, TRTuroni, Srl Forlì, Italy) on JF umbrellas (46, 47). A penetration test was performed using a three-bars probe (3 × 22 mm) for a total plunger area of 1.98 cm^2^ by operating on samples consisting of radial triangular slices of the JF umbrella. Due to the different thickness of *R. pulmo* umbrella, data were the means of three measurements on three regions of umbrella, such as the inner, medium, and external parts. At least three-quarters of five different JF specimens were evaluated. Firmness values were measured for each time point (days 0, 1, 5, 10, and 10 + 1 days) and expressed as Newton (N).

### Protein Content

Fresh JF samples were homogenized in a Waring® laboratory blender (three pulses for 15 s under refrigerated conditions). Approximately 2 g of each homogenized sample was suspended in 2, 4, or 8 ml of MilliQ water in order to obtain a homogeneous suspension.

Badfrod assay (48) was used to evaluate the total protein content. However, this method was modified and adapted to round bottom 96-well microplate for Infinite 200 PRO microplate reader (Tecan, Mannedorf, Switzerland), using bovine serum albumin (BSA, Sigma-Aldrich, Darmstadt, Germany) as standard (40). Means of at least three measurements from two independent experiments were considered.

### Antioxidant Activity

The antioxidant activity was evaluated in each JF sample by the Trolox Equivalent Antioxidant Capacity (TEAC) method, adapted for the Infinite 200 PRO microplate reader (Tecan, Mannedorf, Switzerland) using the radical cation ABTS•+ and Trolox (Sigma-Aldrich, Darmstadt, Germany) as standard (49, 50). The samples and the standard were assayed under the same conditions as already described in De Domenico et al. (40). The results were expressed as nmol of Trolox Equivalents per gram of fresh weight (nmol TE/g FW), and means of at least three measurements from two independent experiments were considered.

### Lipid Extraction and Fatty Acid Analysis

Total lipids were extracted using the modified method of Bligh and Dyer (51), with some modifications (38). Dried samples (200 mg) were mixed with a total of 15 ml solvent added in this sequence: 6 ml of chloroform: methanol (2:1), 6 ml of chloroform: methanol (2:1) and 3 ml KCl (0.88%). The samples were shaken for 15 s after the addition of each solvent and centrifuged at 5,140 × g for 5 min. The lower phase was set aside, and the upper phase was subjected to further extraction with a solution of chloroform: methanol (2:1, 1 vol.). The lower phase was isolated and added to the first one, and mixed with a solution of methanol: water (1:1, $\frac{1}{4}$ volume). In this case, the lower phase was put aside, dried in the presence of nitrogen flux, and analyzed for lipid composition. Then, fatty acid methyl esters (FAMEs) were obtained using boron trifluoride (BF_3_) according to Leone et al. (38), with some modifications. A part of the total lipid extract (200 μl in hexane) was saponified at 90°C for 20 min with 0.5 M KOH in methanol (3 ml). Sixty-six micrograms of the internal standard (methyl-tricosanoate, Sigma-Aldrich, Darmstadt, Germany) was added before saponification. The fatty acids were methylated by adding 2 ml of 14% BF_3_ in methanol (Sigma-Aldrich, Darmstadt, Germany). The samples were evaporated under a stream of nitrogen and dissolved in 50 μl of hexane, and 1 μl was analyzed by gas chromatography-mass spectrometry (GC-MS). GC–MS analyses were performed using an AGILENT 5977E gas chromatograph (Agilent Technologies, Santa Clara, CA, United States). Separation of compounds was performed on a VF-WAXms (60 m, 0.25 mm i.d., 0.25 mm film thickness, Agilent Technologies, Santa Clara, CA, United States). GC parameters were as follows: the column temperature was maintained at 160°C for 1 min, programmed at 4°C/min to 240°C for 30 min. Helium was used as a carrier gas at a constant flow rate of 1 ml/min. A mass spectrometer was operated in electron impact mode with a scan range of 50–700 m/z. The temperature of MS source and quadrupole was set at 230 and 150°C. Analyses were performed in full-scan mode. Compounds were identified by comparing the retention times of the chromatographic peaks with those of authentic standards (F.A.M.E. Mix C8-C24, Sigma-Aldrich, Darmstadt, Germany) analyzed under the same conditions. MS fragmentation patterns were compared with those of pure compounds, and a mass spectrum database search was performed using the National Institute of Standards and Technology (NIST) MS 98 spectral database. Fatty acid composition was expressed as a percentage of the total fatty acids in each sample, while the total lipid content was expressed both as yield of lipid extraction in 100 g of fresh JF sample (mg/100 g FW) and as a percentage of dry weight (% DW).

### Statistical Analysis

All data represent the mean of three independent replicates (*n* = 3). Statistical analysis was based on one-way analysis of variance. Tukey's *post hoc* method was applied to establish significant differences among the means (*p* < 0.05, *p* < 0.01, and *p* < 0.001). All statistical comparisons were performed using Sigma-Stat, version 3.11 (Systat Software Inc., Chicago, IL, United States).

## Results

### JF Pre-treatment

The JF samples washed with sterile seawater, representing a mixed combination of umbrellas and oral arms, revealed the presence of bacterial pathogens belonging to staphylococci and *Vibrio* spp., even if in a limited number (Table 1). This evidence was observed in the agar selective medium and was confirmed by the molecular identification of 16S rRNA sequence performed on five different colonies for *Vibrio* genus (data not shown).

**Table 1:** Microbiological analyses of jellyfish washed with sterile sea water (JFSW) sample.

**Microorganisms**	**Medium**	**JFSW**	
		**Mean (CFU/g)**	**SD**
TBC	PCA	2.38 × 10^3^	1.94 × 10^2^
*Bacillus* spp.	BCSA	6 × 10^2^	1.15 × 10^1^
H_2_S-producing bacteria	IRON AGAR	3.55 × 10^1^	7.78
Enterobacteriaceae	VRBGA	4.65 × 10^1^	4.73
Coli-Aerogenes Bacteria	VRBA	8.15 × 10^1^	1.20 × 10^1^
Coagulase positive staphylococci	Baird Parker Agar	2.50 × 10^2^	7.07 × 10^1^
Pathogenic staphylococci	MSA	0	0
*Pseudomonas* spp.	Pseudomonas Agar	4.65 × 10^1^	4.95
*Vibrio* spp.	TCBSA	5.2 10^2^	2.15 × 10^2^
Yeasts	DRBC	5.56 × 10^2^	7.92 × 10^1^
Molds		0	0
**Halophilic microorganisms**			
Bacteria	R2A	1.10 × 10^1^	1.32
	Marine Agar	5.19 × 10^2^	3.68 × 10^1^
Yeasts	sCMA	1.40 × 10^1^	5.66
	sSDA	1.95 × 10^2^	2.83 × 10^1^
	R2A	2.93 × 10^2^	3.18 × 10^1^
	Marine Agar	8.50 × 10^1^	7.07
Molds	sCMA	1.42 × 10^2^	9.90
	sSDA	2.00 × 10^2^	2.83 × 10^1^
	R2A	1.48 × 10^3^	1.19 × 10^2^
	Marine Agar	3.95 × 10^3^	7.16 × 10^1^

*TBC, total bacterial count at 30°C; CFU, colony-forming unit; JFSW, jellyfish washed with sterile sea water; sCMA, saline corn meal agar; sSDA, saline sabouraud dextrose agar*.

The total bacteria count and the level of *Bacillus* spp. were relatively low (2.4 × 10^3^ and 6 × 10^2^ CFU/g, respectively), whereas negligible counts of hydrogen sulfide-producing bacteria (such as *Pseudomonas* spp. and possibly *Shewanella putrefacens*/*Aereomonas hydrophila*), of coli-aerogenes bacteria and of yeasts were reported. The possible presence of halophilic/halotolerant microorganisms associated with the JFSW samples was tested on saline media (added with artificial seawater). Both bacteria and yeasts/molds were detected with some differences derived from the medium used. Marine agar and R2A seemed more permissive than the classic media (corn meal agar and sabouraud dextrose agar) added with sea salts for yeasts and molds (Table 1).

### JFSW Treatment for Structuring and Stabilization

In this study, a preliminary step was performed by creating an up-to-date overview of the compounds that are currently used as food additives, with the function of firming agents, acidity regulator, stabilizers, and also cross-checking information on their possible use in EU, USA, Australia, and New Zealand (Supplementary Table 1).

Calcium acetate, calcium lactate, and calcium citrate were chosen as brining and firming agents to perform a time course experiment for the treatment of the JF samples washed with sterile seawater. All the three treatments used here were able to limit the growth or eliminate completely the presence of the tested microbial species by 10-day incubation at 4°C (Tables 2A–C). After 5 days of treatment, calcium lactate and calcium citrate (Tables 2B,C) were able to reduce significantly, or eliminate, the presence of tissue-associated microorganisms. However, a slight increase in total bacterial, Enterobacteriaceae, and *Pseudomonas* spp. counts, after 10 days of treatment, was registered. Furthermore, the levels of halophilic bacterial species were limited by both calcium lactate and calcium citrate treatments for up to 5 days, whereas an increase in bacteria was reported against a significant reduction of yeasts and molds after 10 days of treatment. Calcium acetate soaking (Table 2A) showed a slower action, since it was less efficient in lowering microbial presence after 5 days but enhanced its effect after 10 days of treatment, even in the case of halophilic microorganisms.

**Table 2A:** Microbiological analyses during time course treatment of JFSW with calcium acetate brines at 1, 5, and 10 days.

**JFSW treatment on Calcium Acetate brines at 4** ^****°****^ **C**									
**Microorganisms**	**Medium**	**5 days**		**10 days**		**10 d** **+** **1d Fa**		**10 d** **+** **1d R**	
		**Mean (CFU/g)**	**SD**	**Mean (CFU/g)**	**SD**	**Mean (CFU/g)**	**SD**	**Mean (CFU/g)**	**SD**
TBC	PCA	1.45 × 10^2^ (a)	6.08	6.09 × 10^3^ (b)	1.63 × 10^1^	8.13 × 10^3^ (c)	4.60 × 10^1^	5.72 × 10^3^ (d)	1.17 × 10^2^
*Bacillus* spp.	BCSA	0	0	0	0	0	0	0	0
H_2_S-producing bacteria	IRON AGAR	2.15 × 10^1^ (a)	2.12	1.80 × 10^1^ (a)	2.83	3.60 × 10^1^ (b)	8.49	1.09 × 10^3^ (c)	9.90
Enterobacteriaceae	VRBGA	1.38 × 10^3^ (a)	4.53 × 10^2^	1.90 × 10^1^ (b)	3.1	3.80 × 10^2^ (c)	2.00 × 10^1^	2.88 × 10^2^ (c)	7.25 × 10^1^
Coli-Aerogenes Bacteria	VRBA	1.93 × 10^2^ (a)	1.62 × 10^1^	1.96 × 10^3^ (b)	1.26 × 10^2^	3.05 × 10^2^ (c)	1.50 × 10^1^	3.03 × 10^2^ (c)	1.75 × 10^1^
Coagulase positive staphylococci	Baird Parker Agar	0	0	0	0	0	0	0	0
Pathogenic staphylococci	MSA	0	0	0	0	0	0	0	0
*Pseudomonas* spp.	Pseudomonas Agar	1.10 × 10^1^ (a)	1.15	2.90 × 10^1^ (a)	2.05	2.55 × 10^2^ (b)	2.12 × 10^1^	2.75 × 10^2^ (b)	4.95 × 10^1^
*Vibrio* spp.	TCBSA	0	0	0	0	0	0	0	0
Yeast	DRBC	0	0	0	0	0	0	0	0
Molds		0	0	0	0	0	0	0	0
**Halophilic microorganisms**									
Bacteria	R2A	2.48 × 10^3^ (a)	2.45 × 10^1^	0 (b)	0	1.80 × 10^3^ (c)	2.47 × 10^1^	3.90 × 10^3^ (d)	1.45 × 10^2^
	Marine Agar	2.59 × 10^3^ (a)	9.76 × 10^1^	9.58 × 10^3^ (b)	3.54 × 10^1^	1.98 × 10^3^ (c)	5.30 × 10^1^	3.24 × 10^3^ (d)	1.20 × 10^2^
Yeasts	sCMA	0	0	0	0	0	0	0	0
	sSDA	7.18 × 10^2^(a)	1.06 × 10^1^	0 (b)	0	0 (b)	0	0 (b)	0
	R2A	1.10 × 10^1^ (a)	1.71	0 (b)	0	0 (b)	0	0 (b)	0
	Marine Agar	0	0	0	0	0	0	0	0
Molds	sCMA	0	0	0	0	0	0	0	0
	sSDA	0	0	0	0	0	0	0	0
	R2A	0	0	0	0	0	0	0	0
	Marine Agar	1.15 × 10^1^ (a)	2.12	0 (b)	0	0 (b)	0	0 (b)	0

*JF were then washed with drinking water and incubated for 1 day at 4°C with phenolic compounds: ferulic acid (10 d + 1d Fa) and rutin (10 d + 1d R). The different letters in line indicate significant differences between the samples (p < 0.05)*.

*TBC, total bacterial count at 30°C; CFU, colony-forming unit; JFSW, jellyfish washed with sterile sea water; Fa, ferulic acid; R, rutin; sCMA, saline corn meal agar; sSDA, saline sabouraud dextrose agar*.

**Table 2B:** Microbiological analyses during time course treatment of JFSW with calcium citrate brines at 1, 5, and 10 days.

**JFSW treatment on Calcium Citrate brines at 4** ^****°****^ **C**									
**Microorganisms**	**Medium**	**5 days**		**10 days**		**10 d** **+** **1d Fa**		**10 d** **+** **1d R**	
		**Mean (CFU/g)**	**SD**	**Mean (CFU/g)**	**SD**	**Mean (CFU/g)**	**SD**	**Mean (CFU/g)**	**SD**
TBC	PCA	6.15 × 10^1^ (a)	2.12	5.05 × 10^3^ (b)	3.89 × 10^1^	3.74 × 10^3^ (c)	8.63 × 10^1^	5.92 × 10^3^ (d)	1.17 × 10^2^
*Bacillus* spp.	BCSA	0	0	0	0	0	0	0	0
H_2_S-producing bacteria	IRON AGAR	0 (a)	0	4.75 × 10^1^ (b)	3.54	1.10 × 10^1^ (a,b)	2.36	3.90 × 10^2^ (c)	4.24 × 10^1^
Enterobacteriaceae	VRBGA	2.90 × 10^1^ (a)	1.12	8.80 × 10^1^ (b)	2.27	6.93 × 10^2^ (c)	1.52 × 10^1^	1.68 × 10^2^ (d)	2.15 × 10^1^
Coli-Aerogenees Bacteria	VRBA	0 (a)	0	5.60 × 10^1^ (b)	6.3	2.81 × 10^2^ (c)	3.10 × 10^1^	1.40 × 10^2^ (d)	1.00 × 10^1^
Coagulase positive staphylococci	Baird Parker Agar	0	0	0	0	0	0	0	0
Pathogenic staphylococci	MSA	0	0	0	0	0	0	0	0
*Pseudomonas* spp.	Pseudomonas Agar	0 (a)	0	1.90 × 10^1^ (b)	3.05	6.65 × 10^1^ (c)	4.95	2.75 × 10^1^ (d)	3.54
*Vibrio* spp.	TCBSA	0	0	0	0	0	0	0	0
Yeast	DRBC	0	0	0	0	0	0	0	0
Molds		0	0	0	0	0	0	0	0
**Halophilic microorganisms**									
Bacteria	R2A	7.90 × 10^1^ (a)	1.75	8.63 × 10^2^ (b)	9.90	1.41 × 10^3^ (c)	1.60 × 10^2^	3.64 × 10^3^ (d)	1.10 × 10^2^
	Marine Agar	4.10 × 10^1^ (a)	3.23	4.05 × 10^3^ (b)	6.93 × 10^1^	1.76 × 10^3^ (c)	3.43 × 10^2^	4.38 × 10^3^ (b)	5.89 × 10^2^
Yeast	sCMA	0	0	0	0	0	0	0	0
	sSDA	3.15 × 10^1^ (a)	2.12	0 (b)	0	2.03 × 10^2^ (c)	1.06 × 10^1^	2.80 × 10^1^ (a)	1.13 × 10^1^
	R2A	0	0	0	0	0	0	0	0
	Marine Agar	0	0	0	0	0	0	0	0
Molds	sCMA	0	0	0	0	0	0	0	0
	sSDA	0	0	0	0	0	0	0	0
	R2A	0	0	0	0	0	0	0	0
	Marine Agar	0	0	0	0	0	0	0	0

*JF were then washed with drinking water and incubated for 1 day at 4°C with phenolic compounds: ferulic acid (10 d + 1d Fa) and rutin (10 d + 1d R). The different letters in line indicate significant differences between the samples (p < 0.05)*.

*TBC, total bacterial count at 30°C; CFU, colony forming unit; JFSW, jellyfish washed with sterile sea water; Fa, ferulic acid; R, rutin; sCMA, saline corn meal agar; sSDA, saline sabouraud dextrose agar*.

**Table 2C:** Microbiological analyses during time course treatment of JFSW in calcium lactate brines at 1, 5, and 10 days.

**JFSW treatment on Calcium Lactate brines at 4** ^****°****^ **C**									
**Microorganisms**	**Medium**	**5 days**		**10 days**		**10 d** **+** **1d Fa**		**10 d** **+** **1d R**	
		**Mean (CFU/g)**	**SD**	**Mean (CFU/g)**	**SD**	**Mean (CFU/g)**	**SD**	**Mean (CFU/g)**	**SD**
TBC	PCA	7.60 × 10^1^ (a)	5.66	4.18 × 10^3^ (b)	2.58 × 10^2^	1.30 × 10^4^ (c)	1.61 × 10^3^	1.09 × 10^4^ (d)	4.24 × 10^2^
*Bacillus* spp.	BCSA	0	0	0	0	0	0	0	0
H_2_S-producing bacteria	IRON AGAR	0 (a)	0	0 (a)	0	1.98 × 10^2^ (b)	2.47 × 10^1^	3.74 × 10^2^ (c)	6.22 × 10^1^
Enterobacteriaceae	VRBGA	0 (a)	0	5.00 × 10^2^ (b)	2.30 × 10^1^	3.61 × 10^2^ (c)	4.10 × 10^1^	1.39 × 10^3^ (d)	2.63 × 10^2^
Coli-Aerogenes Bacteria	VRBA	0 (a)	0	5.75 × 10^1^ (a)	5.83	3.33 × 10^2^ (b)	4.75 × 10^1^	1.06 × 10^3^ (c)	4.24 × 10^2^
Coagulase positive staphylococci	Baird Parker Agar	0	0	0	0	0	0	0	0
Pathogenic staphylococci	MSA	0	0	0	0	0	0	0	0
*Pseudomonas* spp.	Pseudomonas Agar	0 (a)	0	1.25 × 10^1^ (a)	3.54	1.73 × 10^2^ (b)	1.06 × 10^1^	6.75 × 10^2^ (c)	3.54 × 10^1^
*Vibrio* spp.	TCBSA	0	0	0	0	0	0	0	0
Yeast	DRBC	0	0	0	0	0	0	0	0
Molds		0	0	0	0	0	0	0	0
**Halophilic microorganisms**									
Bacteria	R2A	2.90 × 10^1^ (a)	3.32	0 (a)	0	6.41 × 10^3^ (b)	1.27 × 10^2^	1.35 × 10^4^ (c)	2.12 × 10^3^
	Marine Agar	4.85 × 10^1^ (a)	2.12	4.10 × 10^3^ (b)	1.44 × 10^2^	1.09 × 10^4^ (c)	1.45 × 10^2^	1.17 × 10^4^ (d)	4.24 × 10^2^
Yeast	sCMA	0	0	0	0	0	0	0	0
	sSDA	0 (a)	0	0 (a)	0	7.60 × 10^1^ (b)	6.53	0 (a)	0
	R2A	1.90 × 10^1^ (a)	1.76	0 (b)	0	0 (b)	0	0 (b)	0
	Marine Agar	0	0	0	0	0	0	0	0
Molds	sCMA	0	0	0	0	0	0	0	0
	sSDA	0 (a)	0	0 (a)	0	1.10 × 10^1^ (b)	1.52	0 (a)	0
	R2A	0	0	0	0	0	0	0	0
	Marine Agar	0	0	0	0	0	0	0	0

*JF were then washed with drinking water and incubated for 1 day at 4°C with phenolic compounds: ferulic acid (10 d + 1d Fa) and rutin (10 d + 1d R). The different letters in line indicate significant differences between the samples (p < 0.05)*.

*TBC, total bacterial count at 30°C; CFU, colony forming unit; JFSW, jellyfish washed with sterile sea water; Fa, ferulic acid; R: rutin; sCMA, saline corn meal agar; sSDA, saline sabouraud dextrose agar*.

**Figure 1:**
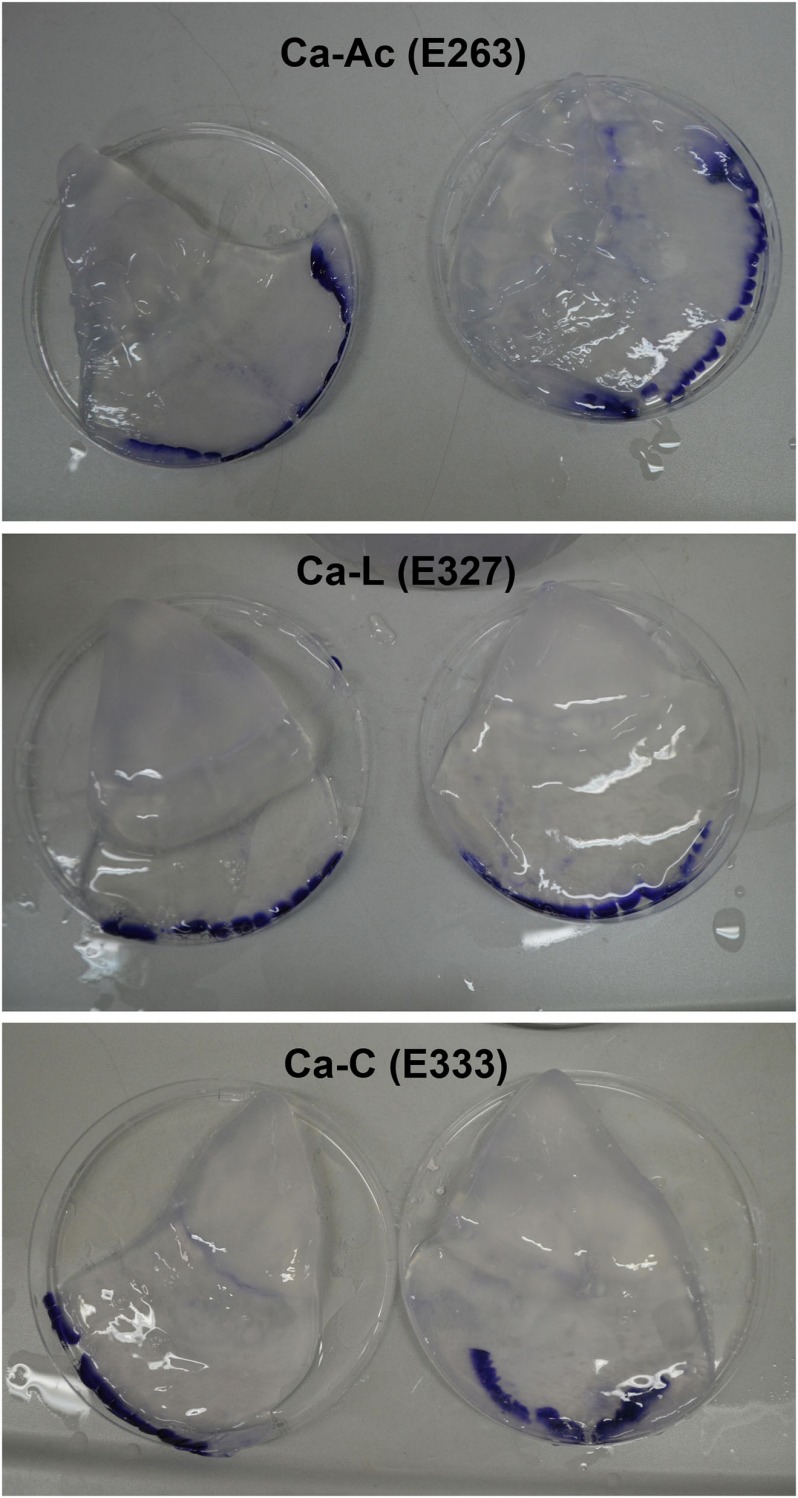
Jellyfish products obtained by the new proposed method using calcium salts as thickening and stabilizing agents. Ca-Ac, calcium acetate; Ca-L, calcium lactate; Ca-C, calcium citrate.

All three treatments produced an increase in texture values after 5 days. Incubations in calcium citrate and lactate showed doubled values in firmness with a slight decrease after 10 days of treatment, whereas JF treated with calcium acetate showed a slower but continuous texture increase until the end of the time-course experiment (Table 3A). In particular, an ~1.8-fold increase in the texture of calcium citrate-treated JF and an ~1.7-fold increase in calcium lactate-treated JF were observed on day 5, with respect to the starting values (0). On day 10, the calcium acetate-treated JF revealed a texture value about 1.3-fold higher than the starting value measured on day 0 (Table 3A). The starting pH value of JF tissue in the absence of brine (pH 7.1) decreased in the presence of calcium-acetate and citrate just after in 1 day-incubation, and it was maintained around 5 after 5 days of treatment, whereas higher pH levels were obtained in calcium lactate brines (Table 3B). Calcium acetate was the only solution able to keep the pH around 5 after 10 days of treatment, even after treatments with phenolic acids (Table 3B). However, all the measured pH values were acceptable to guarantee the safety of the semi-finished products (Tables 2A–C). Lower salinity levels were observed in all the treated samples just after the 1st day of treatment (Table 3C). However, the calcium acetate brine produced undesirable effects on JF consistency by damaging the margins to the extent of punching the tissue in the umbrellas. Figure 1 shows a picture of the semi-finished products obtained with the three methods proposed. All the treatments were also carried out on frozen JFSW, which were stored at −80°C for at least 48 h and then thawed on ice (0°C) overnight before being soaked in the brines. The obtained results, in terms of safety and quality traits, were comparable to those of freshly treated JFSW (data not shown).

**Table 3A:** Texture values during time course treatment of JFSW in brines with calcium acetate, citrate, and lactate at 1, 5, and 10 days.

**Texture (N)** (**Mean** **±** **SD)**						
**Treatment**	**0**	**1 day**	**5 days**	**10 days**	**10d+1d Fa**	**10d+1d R**
Calcium acetate	−67.2 ± 4 ^a^	−70 ± 4 ^a^	−87 ± 5 ^b^	−90 ± 5 ^b^	−98 ± 8 ^b^	−88 ± 5 ^b^
Calcium citrate	−66.4 ± 4 ^a^	−80 ± 5 ^b^	−119 ± 5 ^c^	−92 ± 4 ^d^	−102 ± 7 ^e^	−101 ± 5 ^e^
Calcium lactate	−65.6 ± 4 ^a^	−88 ± 4 ^b^	−109 ± 4 ^c^	−91 ± 6 ^b^	−104 ± 6 ^c^	−99 ± 4 ^b,c^

*JF were then washed with drinking water and incubated for 1 day at 4°C with phenolic compounds (10 d + 1d; Fa: ferulic acid and R: rutin). The different letters in line indicate significant differences between the samples (p < 0.05)*.

**Table 3B:** pH values during time course treatment of JFSW in brines with calcium acetate, citrate, and lactate at 1, 5, and 10 days.

**pH**						
**Treatment**	**0**	**1 day**	**5 days**	**10 days**	**10d+1d Fa**	**10d+1d R**
Calcium acetate	7.10	5.15	5.42	5.16	5.42	5.40
Calcium citrate	7.10	5.08	4.89	7.05	6.95	7.15
Calcium lactate	7.10	6.40	7.03	6.17	6.97	6.84

*JF were then washed with drinking water and incubated for 1 day at 4°C with phenolic compounds (10 d + 1d Fa: ferulic acid and R: rutin)*.

**Table 3C:** Salinity values during time course treatment of JFSW in brines with calcium acetate, citrate, and lactate at 1, 5, and 10 days.

**Salinity (%)**						
**Treatment**	**0**	**1 day**	**5 days**	**10 days**	**10d+1d Fa**	**10d+1d R**
Calcium acetate	4.50	2.80	3.00	3.50	2.00	2.00
Calcium citrate	4.50	2	2.00	2.70	1.20	1.20
Calcium lactate	4.50	3.00	2.80	3.50	2.50	2.50

*JF were then washed with drinking water and incubated for 1 day at 4°C with phenolic compounds (10 d + 1d Fa: ferulic acid and R: rutin)*.

Furthermore, additional steps, freezing (at −80°C) and thawing the samples previously treated with calcium salts, allowed for the achievement of further reduction in their microbial load, and kept the quality and sensory characteristics comparable to those of the samples before being frozen and thawed (data not shown).

### JF Treatment With Phenolics as Collagen Cross-Linkers

Here, the use of phenolic compounds as natural cross-linking agents for protein-based matrices, and to improve the antioxidant activity of JF tissue previously treated with calcium salt was explored. The concentration of the phenolic compounds acting as reticulating agents was empirically set at 2 mg/g collagen content in JF tissue, a value close to the minimum quantities used by Cao et al. (44). This concentration was selected, since the scope of the experiment was to verify the effects of phenolic compounds on the whole tissue texture and their capacity to improve the antioxidant capability of the JF tissues without producing edible films. Bacteria were detectable in all the samples, both in the presence of rutin and ferulic acid (Tables 2A–C). Among halophilic microorganisms, a limited number of molds were detected in the samples treated with calcium lactate and phenolic compounds (both ferulic acid and rutin), whereas yeasts were found in both samples, regardless if they were treated with ferulic acid-calcium citrate and lactate samples or with rutin-calcium citrate. The results total bacterial and Enterobacteriaceae counts were acceptable for the samples first treated with calcium citrate, lactate, and acetate, and then with ferulic acid and rutin; whereas they were satisfactory for *Vibrio* spp., *Bacillus* spp., coagulase-positive staphylococci, and yeasts. After the treatment with both ferulic acid and rutin, the texture was maintained or increased slightly compared with the values of the same samples treated with the three brines on day 10 (Tables 3A,B). Furthermore, the addition of the phenolic compounds did not affect the pH of the samples, which was the same as the pH of the samples treated only with calcium salts for 10 days (Table 3B). Finally, the salinity levels of the samples treated with phenolic compounds were lower than values measured on the 10^th^ day in the corresponding samples that were not treated with the phenolic compounds (Table 3C).

### Effect of Brine Treatment on JF Protein Content

**Figure 2:**
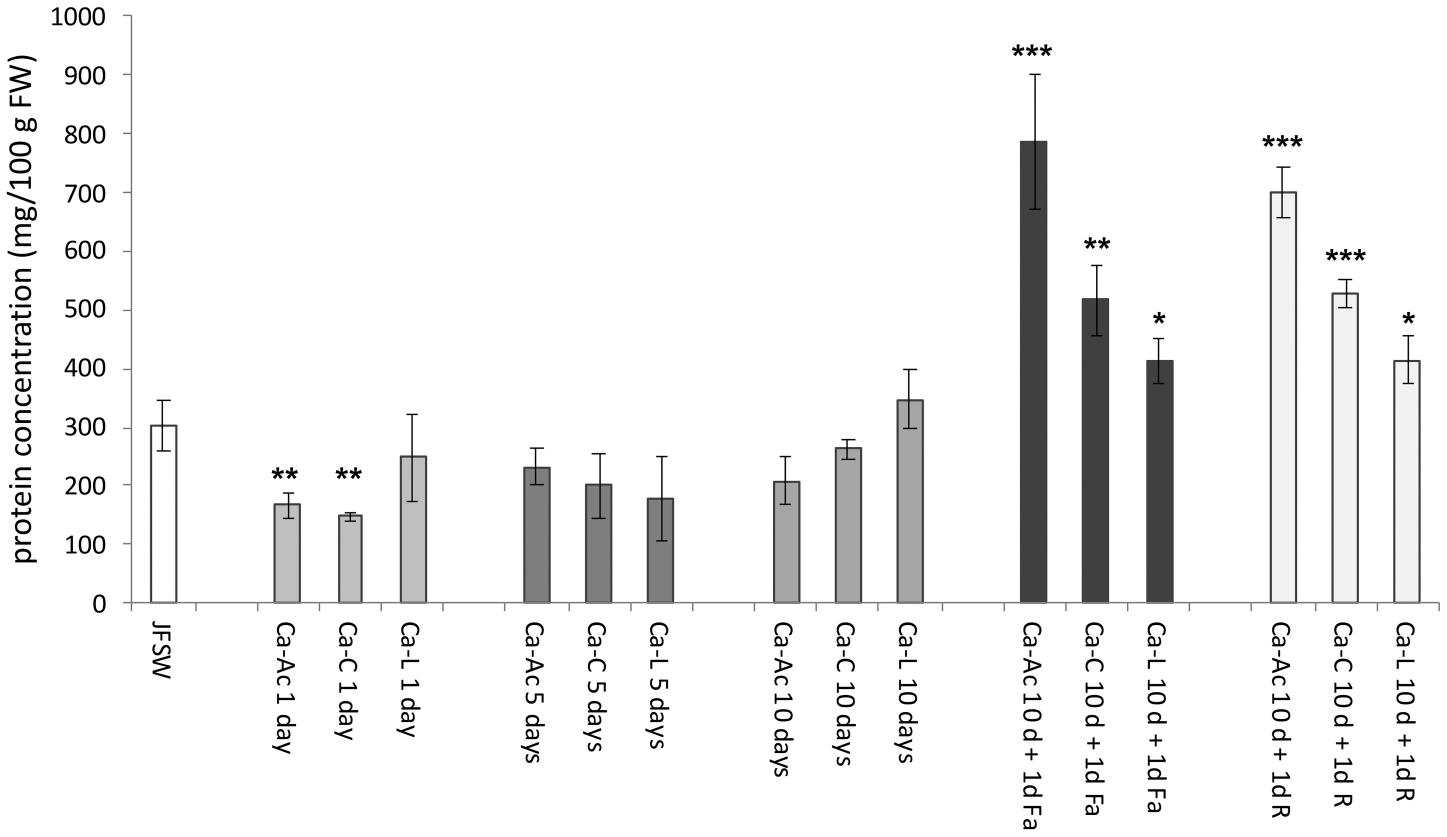
Protein concentration in JF samples. Protein contents expressed as mg per 100 g of FW (mg/100 g FW) in control [jellyfish washed with sterile sea water (JFSW)] and in the different treatments used: Ca-Ac (calcium acetate), Ca-C (calcium-citrate), Ca-L (calcium lactate) for 1, 5, and 10 days; Ca-Ac 10 d + 1d Fa (ferulic acid added to JF already treated for 10 days with Ca-Ac), Ca-C 10 d + 1d Fa (ferulic acid added to JF already treated for 10 days with Ca-C), Ca-L 10 d + 1d Fa (ferulic acid added to JF already treated for 10 days with Ca-L), Ca-Ac 10 d + 1d R (rutin added to JF already treated for 10 days with Ca-Ac), Ca-C 10 d + 1d R (rutin added to JF already treated for 10 days with Ca-C), Ca-L 10 d + 1d R (rutin added to JF already treated for 10 days with Ca-L). Values are the means of three independent measurements, ± standard deviation. ANOVA statistic test, followed by Tukey's multiple comparison post-test, was performed to compare each treatment in each incubation condition (1, 5, and 10 days, and 10 d + 1d Fa and 10 d + 1d R) with the control JFSW and between each of them (*p < 0.05, **p < 0.01, and ***p < 0.001).

The protein content was evaluated using the Bradford assay (48) on the samples obtained by homogenization of untreated and brine-treated JF specimens suitably diluted in water. Raw *R. pulmo* washed with sterile seawater (JFSW) contained about 302 mg of proteins per 100 g of fresh weight (Figure 2). JF treated with calcium salts showed a decrease in protein concentration on day 1 of treatment in the calcium acetate and citrate samples (Ca-Ac 1d and Ca-C 1d), while no significant change in protein concentration was evident in all the other samples until 10 days as compared with control (JFSW). All the three samples treated with calcium salts and either ferulic acid (Ca-Ac 10 d +1d Fa, Ca-C 10 d + 1d Fa and Ca-L 10 d + 1d Fa) or rutin (Ca-Ac 10 d + 1d R, Ca-C 10 d + 1d R and Ca-L 10 d + 1d R) revealed a statistically significant increase in protein concentration as compared with the untreated samples (JFSW), and among them (Figure 2). In fact, an increase in protein concentration of about 1.5 times was found in the samples treated with Ca-lactate or Ca-citrate both added with phenolic acids vs. control (JFSW), while an increase of up to 2.5-fold was found in the samples treated with Ca-acetate and either ferulic acid or rutin vs. control (JFSW).

### Effect of Brine Treatment on Antioxidant Activity

**Figure 3:**
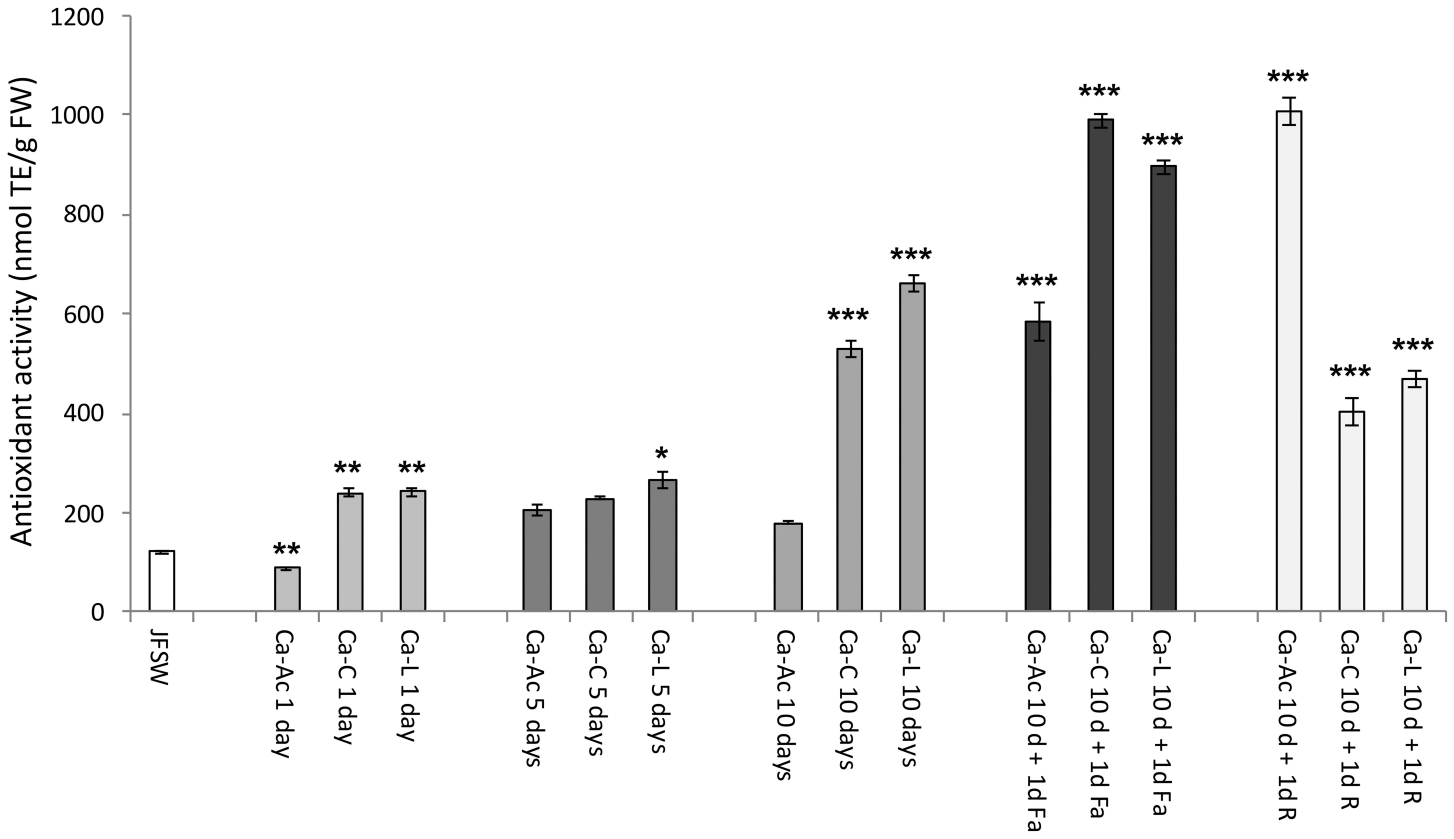
Antioxidant activity in JF samples. Antioxidant activity in control (JFSW) and in the different treatments used: Ca-Ac (calcium acetate), Ca-C (calcium-citrate), Ca-L (calcium lactate) for 1, 5, and 10 days; Ca-Ac 10 d + 1d Fa (ferulic acid added to JF already treated for 10 days with Ca-Ac), Ca-C 10 d + 1 d Fa (ferulic acid added to JF already treated for 10 days with Ca-C), Ca-L 10 d + 1d Fa (ferulic acid added to JF already treated for 10 days with Ca-L), Ca-Ac 10 d + 1 d R (rutin added to JF already treated for 10 days with Ca-Ac), Ca-C 10 d + 1 d R (rutin added to JF already treated for 10 days with Ca-C), Ca-L 10 d + 1 d R (rutin added to JF already treated for 10 days with Ca-L). Mean values of three independent measurements. Antioxidant activity is expressed as nmol of TE per g fresh weight (nmol TE/g FW) ± standard deviation. ANOVA statistic test, followed by Tukey's multiple comparison post-test, was performed to compare each treatment in each incubation condition (1, 5, and 10 days, and 10 d + 1d Fa and 10 d + 1d R) with the control JFSW and between each of them (*p < 0.05, **p < 0.01, and ***p < 0.001).

The antioxidant activity (AA) was evaluated in the JF samples and expressed as nmol of Trolox equivalent for g of fresh weight (nmol TE/g FW, Figure 3). An increase in AA was reported in all the JF samples treated with the calcium salt brines at different times of incubation (1, 5, and 10 days) as compared with the untreated control (JFSW), with the exception of Ca-Ac (Figure 3). JF tissues treated with Ca-C and Ca-L showed a significant time-dependent increase in AA, reaching the highest values on the 10^th^ day of incubation, which can be mainly related to dehydration and possible concentration of the antioxidant compounds during the process, as demonstrated by expressing the AA per mg of protein (data not shown).

As expected, a significant increase in AA was detected in all the JF samples treated for 10 days with the three different calcium salts in the presence of both rutin and ferulic acid. In particular, the highest AA values were recorded in the samples treated with ferulic acid after brining in calcium citrate and calcium lactate (Ca-C 10 d + 1d Fa and Ca-L 10 d + 1d Fa) and the ones treated with rutin after the addition of calcium acetate brine (Ca-Ac 10 d + 1d R). However, a high AA was also found in the samples incubated with calcium acetate and then with ferulic acid (Ca-Ac 10 d + 1d Fa).

### Effect of Brine Treatment on JF Fatty Acid Composition

The fatty acid (FA) composition was expressed as a percentage and was estimated in raw JF samples washed with sterile seawater (day 0) and in all the JF samples treated with the three calcium salts on days 1, 5, and 10 of treatment (Table 4). In the JFSW control sample, the saturated fatty acids (SFAs) accounted for half of the total fatty acid content (FA, about 51%), followed by polyunsaturated fatty acids (PUFAs, about 40%), and a moderate amount of monounsaturated fatty acids (MUFAs, 10% of the total FAs). In all the samples, an increase in the percentage of the SFA content was observed, overtime after 1, 5, and 10 days, from the starting value of about 50% up to about 70–90% of the total FA content. Lower amounts of myristic (C14:0) and margaric (C17:0) acids were detected in all the treated samples, with values lower than those in untreated JFSW in general. The presence of other SFAs, such as pentadecanoic acid (C15:0), non-adecanoic acid (C19:0), and lignoceric acid (C24:0), was also detected, in particular, after 5 days of treatment. Among the samples treated with the calcium salts, the lowest SFA content (about 72%) was found in the JF products treated for 10 days with calcium lactate, in which a high PUFA percentage was also detected. Among the SFAs, palmitic (C16:0) and stearic (C18:0) acids were the most represented ones, whose percentages in the JFSW were about 23 and 17%, respectively. The content of these two SFAs increased in the brine-treated samples, with values registered between 26 and 47%. In all the treated JF samples, oleic acid (C18:1) was not detected, while an increase in isooleic acid (C18:1 *trans*-10) was registered, as the only MUFA present, which is the product of the reaction of oleic acid skeletal isomerization reaction. Total MUFA content generally decreased in all the brine-treated JF samples, except for the samples treated with Ca-Ac and Ca-L for 5 days. In this last sample, MUFA species such as palmitoleic acid (C16:1) and vaccenic acid (C18:1 cis-11) were preserved. Furthermore, total PUFA content was reduced with almost all the treatments, except for the JF samples treated with Ca-L for 10 days, probably due to a high value of AA as described above (Figure 3). Consequently, it was observed that the conditions of the Ca-L treatment for 5 days were more favorable in preserving the diversity of all the PUFA species, since linoleic (C18:2) and linolenic acids (C18:3) were still detectable in a content comparable with that in the untreated control JFSW. In all the other treatments, the PUFA species initially found in the JFSW control were even significantly completely reduced, probably because of the occurrence of lipid oxidation processes.

**Table 4:** Comparison of the fatty acid composition of jellyfish *R. pulmo* treated with brines containing different calcium salts at 1, 5, and 10 days treatment.

	**Fatty Acids (%)**									
	**0**	**1 day**			**5 days**			**10 days**		
	**JFSW**	**Ca-Ac**	**Ca-C**	**Ca-L**	**Ca-Ac**	**Ca-C**	**Ca-L**	**Ca-Ac**	**Ca-C**	**Ca-L**
**Saturated FA (SFA)**										
Myristic acid C_14:0_	4.4 ± 0.4	2.4 ± 0.2	0.7 ± 0.1	2.2 ± 0.2	—	2.6 ± 0.2	3.6 ± 0.3	2.6 ± 0.3	2.0 ± 0.2	3.4 ± 0.3
Pentadecanoic acid C_15:0_	0.8 ± 0.1	—	—	—	5.0 ± 0.4	1.3 ± 0.1	0.8 ± 0.1	—	—	3.6 ± 0.4
Palmitic acid C_16:0_	22.8 ± 2.3	47.3 ± 0.5	44.9 ± 4.5	39.7 ± 0.4	34.7 ± 4.0	39.4 ± 3.8	35.4 ± 3.3	39.4 ± 4.0	38.3 ± 3.8	33.4 ± 3.3
Margaric acid C_17:0_	4.0 ± 0.4	—	1.7 ± 0.2	2.4 ± 0.2	6.6 ± 0.2	3.6 ± 0.2	2.4 ± 0.2	1.9 ± 0.2	1.5 ± 0.2	2.1 ± 0.2
Stearic acid C_18:0_	17.4 ± 1.7	39.8 ± 0.4	39.4 ± 4.0	35.1 ± 3.5	43.1 ± 3.9	39.5 ± 4.0	26.1 ± 2.7	38.7 ± 3.9	39.8 ± 4.0	27.2 ± 2.7
Non adecanoic acid C_19:0_	0.6 ± 0.1	—	—	—	—	—	0.6 ± 0.1	—	—	—
Arachidic acid C_20:0_	1.4 ± 0.1	1.3 ± 0.1	1.0 ± 0.1	1.8 ± 0.2	—	3.1 ± 0.2	2.0 ± 0.3	1.5 ± 0.1	2.5 ± 0.2	2.6 ± 0.3
Behenic acid C_22:0_	—	—	—	—	—	—	1.5 ± 0.2	—	—	—
Lignoceric acid C_24:0_	—	—	—	—	—	0.4 ±0.1	11.8 ± 0.2	—	—	—
Total SFA	51.5 ± 5.0	90.7 ± 9.1	87.8 ± 8.8	81.1 ± 8.0	89.4 ± 8.9	89.9 ± 8.9	84.2 ± 8.4	84.1 ± 8.4	84.1 ± 8.4	72.2 ± 7.2
**Monounsaturated FA (MUFA)**										
Palmitoleic acid C_16:1_ (ω7)	3.8 ± 0.4	—	1.6 ± 0.2	—	—	—	2.8 ± 0.3	—	—	—
Oleic acid C_18:1_ (ω9)	4.4 ± 0.5	—	—	—	—	—	—	—	—	—
Isooleic acid C_18:1trans−10_	—	—	3.0 ± 0.3	2.8 ± 0.3	10.6 ± 3.4	3.8 ± 0.4	1.3 ± 0.4	3.6 ± 0.4	3.8 ± 0.4	4.3 ± 0.4
Vaccenic acid C_18:1cis−11_ (ω7)	2.0 ± 0.2	—	—	—	—	—	3.9 ± 0.4	—	—	—
Nervonic acid C_24:1_ (ω9)	—	—	—	—	—	0.4 ± 0.1	—	—	—	—
Total MUFA	10.2 ± 1.0	0	4.6 ± 0.5	2.8 ± 0.3	10.6 ± 3.4	4.2 ± 0.4	8.0 ± 0.7	3.6 ± 0.4	3.8 ± 0.4	4.3 ± 0.4
**Polyunsaturated FA (PUFA)**										
Linoleic acid C_18:2_(ω6)	2.8 ± 0.3	—	—	—	—	—	2.2 ± 0.2	—	—	—
7,10-Octadecenoic acid C_18:2_	—	—	—	—	—	—	—	—	—	2.2 ± 0.2
Linolenic acid C_18:3_ (ω3)	1.3 ± 0.1	—	—	—	—	—	0.9 ± 0.1	—	—	—
Stearidonic acid C_18:4_ (ω3)	1.4 ± 0.1	—	—	—	—	—	—	—	—	2.6 ± 0.3
Eicosadienoic acid C_20:2_(ω6)	—	—	—	—	—	1.5 ± 0.2	—	—	—	—
Arachidonic acid C_20:4_(ω6)	9.6 ± 1.0	3.1 ± 0.3	3.0 ± 0.3	3.8 ± 0.4	—	3.1 ± 0.7	2.4 ± 0.2	5.1 ± 0.5	6.6 ± 0.7	7.7 ± 0.8
Eicosapentaenoic acid C_20:5_ (ω3)	7.2 ± 0.7	3.7 ± 0.4	3.0 ± 0.3	4.2 ± 0.4	—	—	1.2 ± 0.6	4.5 ± 0.5	4.0 ± 0.4	5.8 ± 0.6
Heneicosatetraenoic acid C_21:4_ (ω3)					—	0.6 ± 0.1	—	—	—	—
Docosatetraenoic acid C_22:4_ (ω6)	1.8 ± 0.2	—	—	—	—	—	—	—	—	0.8 ± 0.1
Docosapentaenoic acid C_22:5_ (ω3)	2.0 ± 0.2	—	—	—	—	—	—	—	—	—
Docosahexaenoic acid C_22:6_ (ω3)	12.3 ± 1.2	2.5 ± 0.2	1.7 ± 0.2	8.1 ± 0.8	—	0.7 ± 0.1	1.1 ± 0.4	2.7 ± 0.3	1.5 ± 0.1	4.4 ± 0.4
Total PUFA	38.4 ± 3.8	9.3 ± 0.9	7.6 ± 0.8	16.1 ± 1.6	0	6.5 ± 0.5	7.8 ± 2.4	12.3 ± 1.2	12.1 ± 1.2	23.5 ± 2.4
Σω6	14.2	3.1	3	3.8	—	4.6	4.6	5.1	6.6	8.5
Σω3	24.2	6.2	4.7	12.3	—	1.3	3.2	7.2	5.5	12.8
ω6/ω3	0.6	0.5	0.6	0.3	—	3.5	1.4	0.7	1.2	0.7
Total lipids (% DW)	2.7 ± 0.2	1.2 ± 0.2	5.9 ± 1.2	1.9 ± 0.1	4.6 ± 0.5	3.6 ± 0.4	4.0 ± 0.4	2.0 ± 0.3	1.9 ± 0.2	3.3 ± 0.4
Total lipids (mg/100 g FW)	190.9 ± 18.2	110.0 ± 10.9	492.6 ± 32.5	199.1 ± 15.9	150.9± 15.9	112.0 ± 11.2	135.4 ± 13.6	201.9 ± 22.6	209.4 ± 21.6	345.3 ± 32.8

*Fatty acid composition data are expressed as percentage of the total fatty acids ± standard deviation (SD). JFSW, jellyfish washed with sterile sea water; Ca-Ac, calcium acetate; Ca-C, calcium citrate; Ca-L, calciumlactate*.

Table 5 shows how the addition of the phenolic acids to the JF samples after 10 days of treatment with the calcium salts seemed to improve FA composition both qualitatively and quantitatively. In particular, a very low increase in SFAs (about 60%) was reported in two of the samples treated with rutin (such as Ca-Ac + 1d R and Ca-L + 1d R). On the other hand, MUFA and PUFA composition profiles varied in almost all the applied treatments. Palmitoleic acid (C16:1) was almost preserved in all the brines except for a little decrease in its content (about 1%). Furthermore, the addition of the phenolic acids to the samples treated with calcium lactate helped to prevent the isomerization of oleic acid (C18:1) to isooleic acid (C18:1 tran-10). In fact, oleic acid was still present in the samples treated with Ca-L and phenolic acids; in these terms, ferulic acid (Ca-L+ 1d Fa containing 3.4% oleic acid) showed better results than rutin (Ca-L+ 1d R 2.2% oleic and 1.1% isooleic acid). Phenolic acids also contributed to preserve the variety in PUFA composition. In particular, the addition of rutin (Ca-Ac+ 1d R and Ca-L+ 1d R) allowed for the preservation of quite high levels of PUFA (about 35% of total FA). In fact, although isomerization of linoleic acid (C18:2) into 7,10-Octadecenoic acid (C18:2) and loss of linolenic acid (C18:3) occurred, long-chain PUFAs, such as arachidonic acid (C20:4, ω6), eicosapentaenoic acid (C20:5, ω3), docosatetraenoic acid (C22:4, ω6), docosapentaenoic acid (C22:5, ω3), and docosahexaenoic acid (C22:6, ω3), were still present in almost all the JF samples, especially when Ca-L was used. In particular, in the Ca-L+ 1d R and in Ca-Ac+ 1d R samples, valuable percentages of fatty acids ω6 (such as arachidonic acid) and ω3 (such as eicosapentaenoic acid) were detected.

**Table 5:** Comparison of the fatty acid composition of jellyfish *R. pulmo* treated for 10 days with different calcium salts and then washed with drinking water and incubated for 1 day at 4°C with phenolic compounds.

**Fatty acids (%)**							
	**0**	**10 days**					
	**JFSW**	**Ca-Ac + 1d Fa**	**Ca-C + 1d Fa**	**Ca-L + 1d Fa**	**Ca-Ac + 1d R**	**Ca-C + 1d R**	**Ca-L + 1d R**
**Saturated FA (SFA)**							
Myristic acid C_14:0_	4.4 ± 0.4	5.5 ± 0.6	2.4 ± 0.2	2.6 ± 0.3	3.2 ± 0.3	—	1.7 ± 0.2
Pentadecanoic acid C_15:0_	0.8 ± 0.1	—	—	—	—	—	—
Palmitic acid C_16:0_	22.8 ± 2.3	33.3 ± 3.4	39.5 ± 4.0	33.4 ± 3.3	28.1 ± 2.8	42.9 ± 4.3	29.7 ± 0.3
Margaric acid C_17:0_	4.0 ± 0.4	2.0 ± 0.2	1.2 ± 0.1	1.5 ± 0.2	1.1 ± 0.1	2.7 ± 0.3	1.2 ± 0.1
Stearic acid C_18:0_	17.4 ± 1.7	32.3 ± 3.2	38.1 ± 3.8	34.5 ± 3.4	27.5 ± 0.3	42.1 ± 4.2	26.2 ± 0.3
Non adecanoic acid C_19:0_	0.6 ± 0.1	—	—	—	—	—	—
Arachidic acid C_20:0_	1.4 ± 0.1	2.4 ± 0.2	1.9 ± 0.2	1.3 ± 0.1	0.5 ± 0.1	1.5 ± 0.1	0.6 ± 0.1
Behenic acid C_22:0_	—	2.4 ± 0.2	1.3 ± 0.1	—	0.6 ± 0.1	—	—
Lignoceric aci C_24:0_	—	—	—	—	—	—	—
Total SFA	51.5 ± 5.0	78.0 ± 7.8	84.4 ± 8.4	73.3 ± 7.3	61.0 ± 6.1	89.2 ± 1.0	59.4 ± 0.6
**Monounsaturated FA (MUFA)**							
Palmitoleic acid C_16:1_ (ω7)	3.8 ± 0.4	2.6 ± 0.3	1.3 ± 0.1	1.7 ± 0.2	1.5 ± 0.2	—	1.5 ± 0.2
Oleic acid C_18:1_ (ω9)	4.4 ± 0.5	—	—	3.4 ± 0.3	—	—	2.2 ± 0.2
Isooleic acid C_18:1trans−10_	—	4.6 ± 0.5	3.7 ± 0.4	—	3.2 ± 0.3	—	1.1 ± 0.1
Vaccenic acid C_18:1cis−11_ (ω7)	2.0 ± 0.2	—	—	—	—	—	—
Nervonic acid C_24:1_ (ω9)	—	—	—	—	—	—	—
Total MUFA	10.2 ± 1.0	7.2 ± 0.7	5.0 ± 0.5	5.1 ± 0.5	4.7 ± 0.5	0	4.8 ± 0.5
**Polyunsaturated FA (PUFA)**							
Linoleic acid C_18:2_(ω6)	2.8 ± 0.3	—	—	—	—	—	—
7,10-Octadecenoic acid C_18:2_	—	2.5 ± 0.2	1.6 ± 0.2	1.8 ± 0.2	1.3 ± 0.1	—	1.2 ± 0.1
Linolenic acid C_18:3_ (ω3)	1.3 ± 0.1	—	—	—	—	—	—
Stearidonic acid C_18:4_ (ω3)	1.4 ± 0.1	—	—	—	—	—	—
Eicosadienoic acid C_20:2_(ω6)	—	—	—	—	—	—	—
Arachidonic acid C_20:4_(ω6)	9.6 ± 1.0	5.4 ± 0.4	3.4 ± 0.4	2.2 ± 0.1	16.3 ± 1.6	5.1 ± 0.5	19.7 ± 1.9
Eicosapentaenoic acid C_20:5_ (ω3)	7.2 ± 0.7	3.4 ± 0.3	3.5 ± 0.4	14.4 ± 1.4	11.9 ± 1.1	3.5 ± 0.3	11.6 ± 1.2
Docosatetraenoic acid C_22:4_ (ω6)	1.8 ± 0.2	0.8 ± 0.1	—	0.6 ± 0.1	0.8 ± 0.1	—	—
Docosapentaenoic acid C_22:5_ (ω3)	2.0 ± 0.2	0.6 ± 0.1	—		0.9 ± 0.1	—	0,6 ± 0.1
Docosahexaenoic acid C_22:6_ (ω3)	12.3 ± 1.2	2.3 ± 0.2	1.9 ± 0.2	2.6 ± 0.3	2.9 ± 0.3	2.3 ± 0.2	2.6 ± 0.3
Total PUFA	38.4 ± 3.8	15.0 ± 1.5	10.5 ± 1.0	21.6 ± 2.2	34.1 ± 3.4	10.8 ± 1.1	35.7 ± 0.4
Σω6	14.2	6.2	3.4	2.8	17.1	5.1	19.7
Σω3	24.2	6.3	5.4	17	15.7	5.8	14.8
ω6/ω3	0.6	1.0	0.6	0.2	1.1	0.9	1.3
Total lipids (% DW)	2.7 ± 0.2	3.9 ± 0.6	4.1 ± 1.4	4.4 ± 0.1	3.5 ± 0.4	3.7 ± 0.4	3.0 ± 0.4
Total lipids (mg/100 g FW)	190.9 ± 18.2	331.2 ± 33.4	339.7 ± 22.8	443.7 ± 45.3	313.3 ± 25.4	346.2 ± 34.5	322.2 ± 20.5

*Fatty acid composition data are expressed as percentage of the total fatty acids ± SD. JFSW, jellyfish washed with sterile seawater; Ca-Ac, calcium acetate; Ca-C, calcium citrate; Ca-L, calcium lactate; Fa, ferulic acid; R, rutin*.

As shown in Tables 4, 5, treatments with almost all the three calcium salts result in a ω6/ω3 ratio lower than 1, thus keeping the ratio of omega-6 to omega-3 essential fatty acids (EFA) naturally present in the untreated material unaltered or even improved. Moreover, rutin was not able to preserve the ω6/ω3 ratio lower than 1 in the JF samples previously treated either with Ca-acetate or Ca-lactate (Ca-Ac + 1d R and Ca-L + 1d R, Table 5). Finally, total lipid content in the JF samples varied from 191 mg/100 g FW in the JFSW to around 200–345 mg/100 g FW after 10 days of treatment with the calcium salt brines (Table 4), and to 313–350 mg/100 g FW in the presence of the phenolic acids (Table 5). The results of total lipid content, expressed per DW, indicated that in most of the analyzed treatments and time points, no significant lipid loss was detected when compared with the content in the starting material (JFSW).

## Discussion

**Figure 4:**
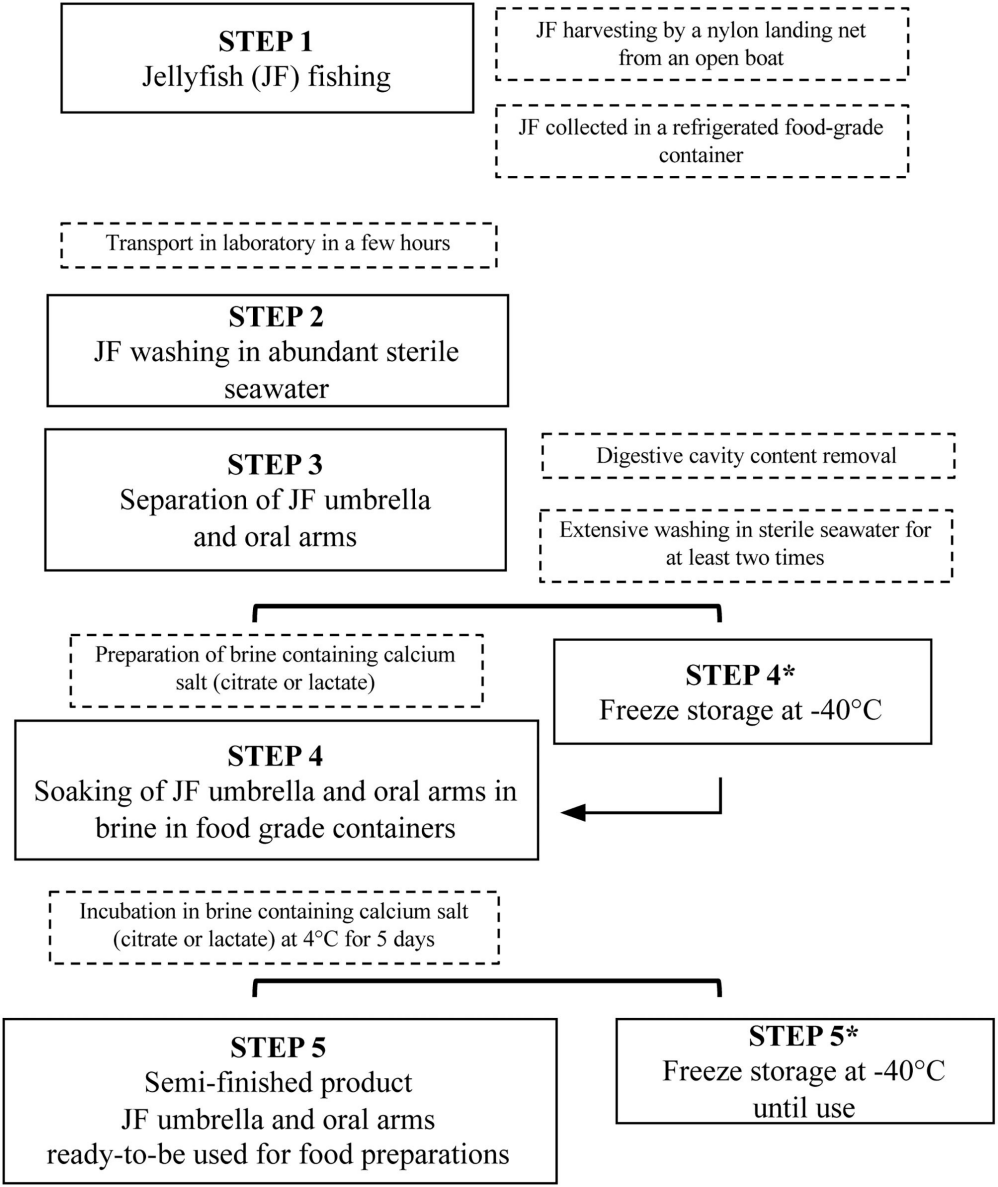
Diagram illustrating the procedure of the new proposed JF treatment method.

This study aimed to set up a new procedure to stabilize and process JF biomass, thus developing a new treatment of JF for food uses, substantially different from the traditional methods used in Asian countries. The main phases proposed for JF preparation are shown in Figure 4, from the starting material to semi-finished food products, and are also reported step-by-step more visually in Supplementary Figure 1.

In the near future, this approach could offer the chance to convert an increasing problem, JF blooms, into a new opportunity for the fishing sector (1). The optimized treatment here proposed aims to produce new evidence that some JF species can be suitable for human consumption, while respecting the safety and quality standards of European, American, and Australian legislations. The use of sterile seawater, already proposed for extensive washing of raw JF (26), was maintained as starting pre-treatment for subsequent JF processing. Although the use of sterile seawater is not applicable for high-volume samples, this condition was chosen in these set-up experiments, because it offers the most permissive environmental conditions to monitor the presence of almost all JF-associated microorganisms. In this study, the parameters chosen to perform JF risk assessment and nutritional characterization were selected among the ones suggested by Raposo et al. (24) and Bleve et al. (26). However, in the set-up of this study, *Salmonella* and *Listeria* were not considered, since they were absent in all previously analyzed JF samples (26); whereas, according to Raposo et al. (24), the screen for *Vibrio* spp. was included as a specific contaminant of marine species.

The data reported in this study demonstrated that the initial microbial charge of JF should still be considerable, even after the elimination of gastric content by dissection of oral arms and umbrellas, and washing with seawater. The presence of bacteria associated to JF body surfaces and mucus secretions was already reported for *R. pulmo* (49) and other JF species [*Aurelia solida* by Kos Kramar et al. (50); *Nemopsisbachei* and *Aurelia aurita* by Daley et al. (51), Weiland-Bräuer et al. (52)]. Moreover, different genera of fungi (such as *Aspergillus, Cladosporium, Purpureocillium*, and *Tilletiopsis*) were isolated from *Nemopilema nomurai* (53). The presence of bacterial genera such as *Stenotrophomonas, Alteromonas, Pseudoalteromonas, Flavobacterium, Coxiella*, and *Vibrio* can represent a potential hazard to humans and other marine organisms (26, 50, 54), even though no pathogens were revealed on both *Catostylustagi* raw material and umbrella-derived commercial snacks (24).

Then, monitoring of bacteria, yeasts, and molds during the different treatment steps of JF-derived products can be useful to reveal microorganisms not yet considered by the established criteria and counting methods for foods enforced in Europe (26). In terms of food safety, other potential microbial halophilic/halotolerant species should be treated as safety hazards. This evidence confirmed the peculiar high-level variation in bacterial associated communities within and among different JF species, this behavior being very different from sponges and bacterioplankton (55). In fact, they should be monitored and their presence should be reduced as a precautionary measure, at least, while waiting for more detailed studies on their possible pathogenic activities. However, as already performed for *Catostylus tagi* (24), the evaluation of hazardous chemical elements (i.e., heavy metal content) and allergenic tests (both on healthy people and patients affected by seafood allergy) on the JF raw material and treated products should be considered as fundamental assays for potentially edible JF-derived products (5). The method proposed here for JF stabilization and treatment is different from the traditional Asiatic procedure and is based on stabilizing agents diverse from alum. The procedure setup provides brines made of calcium acetate, calcium lactate, or calcium citrate solutions for soaking of tissues. This approach varies significantly from the already existing practices for JF treatment, generally based on placing several layers of JF umbrellas singularly overlapped with solid salt and alum mixtures applied directly on the tissue surface (6).

It was demonstrated here that the treatment of JF tissue with calcium citrate, lactate, and acetate was able to stabilize the microbial load and modify the tissue texture. In fact, all the samples treated with any of the three different calcium solutions were considered satisfactory for all tested microbiological parameters: total bacterial count (56), Enterobacteriaceae (57), *Vibrio* spp. (56, 58, 59), *Bacillus* spp. (56), coagulase positive staphylococci (27, 28, 56, 58, 60, 61), and yeasts (61). Compliance with both microbiological and toxicological required parameters is of primary importance in the formulation of a new food. Obviously, the nutritional and organoleptic features are also relevant. The peculiar texture and crunchiness of the JF food product are an organoleptic quality highly appreciated in Asia and strictly depend on the alum treatment. The search for alternative metals to aluminum in the traditional Asian method to obtain a “rubber-like” consistency for JF is currently under consideration (62).

As expected, this study reports that calcium ions are not able to replace alum in collagen cross-linking, and produce the rubber-like structure for JF. This is probably because of the inability of calcium ions to lower pH as efficiently as alum, and the different valence of metal ions (62).

pH values registered after this treatment were in a range between 4.89 and 7.15, thus avoiding the JF tissue dissolution and suggesting that this parameter should not be fundamental for JF maintenance (62). Even though Pedersen et al. (62) demonstrated that calcium ions cannot be used for tanning purposes, the JF samples here treated with calcium salt-based brines showed an increase in texture in all the tested conditions after 5 days, although not crunchy but rather gelatinous-stiff consistency was achieved. This evidence can be explained by the activity of many collagen carbonyl groups that can chelate calcium ions, thus causing the hardening of the soft tissues. The ability of calcium-based food additives (calcium acetate, calcium chloride, calcium lactate) to improve gelation, in particular polymerization, was already demonstrated in the preparation of surimi based on codfish species (63). The calcium salt treatments were also successfully applied to the previously frozen (−80°C) JFSW, since they were able to ensure quality and safety levels comparable with those of freshly treated samples (data not shown). Freezing the JFSW at a very low temperature, by mimicking the application of blast chiller equipment in food companies, can offer the opportunity to store the raw material until use, without loss of its starting quality and sensory characteristics.

Additionally, the method proposed here could be a safe alternative to avoid the use of alum in processing JF tissues. Indeed, due to safety issues related to aluminum intake, alum presence could represent an obstacle to the market of edible JF in EU. In European countries, the law is very restrictive because of possible neurotoxic and cumulative effects of aluminum salts, indicating a TWI of 1 mg aluminum/kg body weight/week (19, 21). These concerns are beginning to be also shared by Asian countries. For example, in Hong Kong, due to the recent issues about the high accumulation of aluminum in the population, the Centre for Food Safety of the Hong Kong Government has expressed an interest in the implementation of strategies to reduce or replace aluminum as an additive in the food preparation process, including JF (64, 65).

In this study, the use of phenolic acids to improve JF processing and the whole quality of JF-based food products is proposed as an innovative idea. In fact, the preliminary data reported here demonstrated that phenol species, such as ferulic acid and rutin, were able to react with JF collagen-rich tissue and improve its mechanical and antioxidant properties. Ferulic acid has low toxicity, is ubiquitous in plants, and is characterized by antioxidant, antimicrobial, anti-cancer, and cholesterol-reducing activities. It is able to form cross-linking interactions in gelatine type-B-form bovine bone (44, 66) and in soy-protein isolated film (67). Similarly, among plant flavonols, rutin was selected as polyphenolic compound, since it enhances mechanical strength and reduces swelling by its ability to form cross-link interactions in type A gelatine (obtained by acid hydrolysis) (68). Interestingly, natural extracts from foods and food industry by-products, such as coffee, grape juice, olives, and other plant sources, could be considered useful to obtain food-safe and food-grade polyphenols for JF treatment.

The data reported in this study demonstrated that treatment with calcium salts after 5 and 10 days did not affect the protein content of the JF samples consistently. Indeed, total protein content in the fresh and treated samples is in accordance with the data previously reported for *R. pulmo* (39). Instead, a further incubation of JF samples, treated for 10 days with calcium salts with phenol compounds (rutin and ferulic acid), led to a significant increase in protein concentration by up to 2–5-fold higher in Ca-Ac 10 d + 1 d Fa and Ca-Ac 10 d + 1d R compared with the unprocessed control (JFSW). This effect could be related to the partial dehydration of the JF tissues and probably to the structural changes that lead to water exclusion. In fact, a texture increase was also recorded in all the treated samples (Table 3A). Similarly, in a study on surimi, the addition of calcium salts reduced the water holding capacity (WHC) of surimi gels, likely because of the activation of endogenous proteinases by Ca^2+^, leading to a network disruption and consequently a lower ability to retain water (69). The good firmness of the products could be due to the action of transglutaminases (TG) as binding agents, possibly previously present in JF tissues. TGs have already been found in other cnidarians (70) and their action to crosslink proteins should be favored in the pH range of 5–8 (71), very close to the brine pH conditions registered here (Table 3B).

The antioxidant activity, which was initially comparable with that reported in a previous study (39), increased in all the obtained calcium salt-treated JF food products, with the exception of the calcium acetate-treated samples, as the incubation time increased. Instead, the use of a natural antioxidant (such as rutin or ferulic acid) proposed here significantly increased the AA in all the samples previously treated with calcium salts, by producing an increase from 3.3 to 8.3-fold higher than in the starting material. Several reactions (oxidation and/or the activation of endogenous proteases) are very common during food processing, and they can be responsible for changes in the food structure. Furthermore, some endogenous proteases (calpains, cathepsins, elastase, and collagenase) were reported to be involved in partial hydrolyzation of proteins with consequent enhancement of their bioactivity, as well as their antioxidant activity, in fish muscle (72) and dry-cured meat products (73). Recent studies have shown a high AA for low molecular-weight JF peptides, derived by enzymatic hydrolysis from *R. pulmo* proteins, such as collagen (40).

Food antioxidants, either endogenous or added during the process, can scavenge free radicals and increase the shelf life of a product, by delaying lipid peroxidation, which is one of the major reasons for the deterioration of food products during storage (74).

Phenol compounds seemed to be also related to the enhancement of the antioxidant activity in the calcium acetate-treated samples and the preservation of polyunsaturated fatty acids. The increased SFA content, observed in almost all the treated samples, especially in the absence of phenolic acids, could be mainly due to lipid oxidation processes. The FA composition of the untreated JFSW, here used as starting point control, was similar to that of the untreated ones and immediately lyophilized the raw JF samples, already analyzed in a previous study by Leone et al. (38). However, lipid peroxidation should lead to the production of new FA species (saturated and unsaturated), derived from the breaking of carbon chains and by the conversion of unsaturated FA to saturated form (75). A significant increase in SFA percentage, mainly for stearic and palmitic acids, together with the concurrent production of new FA species (such as lignoceric acid), and the decrease in some long-chain FA (C20, C22) species of PUFA, were found in JF treated with calcium salts at different incubation times. Nevertheless, the minimal presence of microorganisms registered in the JF samples could be responsible for the hydrogenation of oleic acid into stearic acid and the concurrent bio-hydrogenation of unsaturated fatty acids to trans fatty acids (76). In fact, oleic acid was replaced by high levels of iso-oleic acid, one of its isomeric trans-forms, in most of the calcium salt-treated JF samples. Nevertheless, MUFA and PUFA contents were not significantly affected in the samples treated with phenolic acids. This was probably because of the capacity of phenolic acids to preserve all the different PUFA species from degradation and keep the levels of MUFA such as palmitoleic and oleic acids unaltered, especially in the JF samples treated with calcium lactate. Therefore, the analysis of both FA composition and lipid profile could be possibly considered as a marker to monitor the process and evaluate the quality of JF products during and after the proposed treatment.

For nutritional evaluation of the samples, the ω6/ω3 ratio was also considered. It resulted lower than 1 after almost all the treatments used here, and slightly higher than 1 in some of the three brine conditions proposed here: these results conformed to an improvement in the essential FA ratio initially present in the untreated JFSW. Although the quantities of PUFA reported here are almost negligible compared with the entire mass of JF, the presence of omega-3 FA in levels higher than those of omega-6 ones should give a beneficial contribution against the most common diseases that afflict Western countries, where the average ω6/ω3 ratio of food is ~15–17 (77). In fact, a daily consumption of omega-3 fatty acids and a reduction in the ω6/ω3 ratio to 2.5–5 is currently recommended for the prevention of the majority of cardiovascular diseases (78) and breast cancer (79), and in the treatment of rheumatoid arthritis and asthma (80). Since dietary omega-3 PUFAs and an optimal ω6/ω3 ratio is important for the maintenance of human health, the development of methods able to preserve these compounds during food processing should be considered.

## Conclusion

For the first time, a very promising JF processing procedure was presented here, in accordance with many safety and quality requirements and regulations of Western countries. This new approach, also described in a very recently granted Italian patent and a related European patent (81), can give a contribution to the design of a new food chain based on JF and JF-derived foods, which can be considered in the future as a novel food in Western countries, including EU.

Although the obtained products are very different from the traditional Asian ones, this study provides new insights on the possible production of JF as food for humans by replacing alum as a stabilizing and thickening agent with other salts permitted by the EU regulations. Furthermore, the use of natural antioxidants (such as phenolic compounds) derived from plants and/or by-products of the food industry as additional stabilizing agents is a very promising strategy for producing healthier JF products, and promoting a circular economy model.

## Data Availability Statement

The raw data supporting the conclusions of this article will be made available by the authors, without undue reservation.

## Author Contributions

FR, SD, and GB conduction of the experiments, acquisition, and interpretation of the results. GB and AL work design, data discussion, and manuscript preparation. All authors contributed to the article and approved the submitted version.

## Conflict of Interest

The authors declare that the research was conducted in the absence of any commercial or financial relationships that could be construed as a potential conflict of interest.

## Publisher's Note

All claims expressed in this article are solely those of the authors and do not necessarily represent those of their affiliated organizations, or those of the publisher, the editors and the reviewers. Any product that may be evaluated in this article, or claim that may be made by its manufacturer, is not guaranteed or endorsed by the publisher.

## Supplementary Material

Supplementary Figure 1 Steps of JF processing (corresponding to steps 2 to 5 shown in Figure 4) to obtain semi-finished products for human food production.

Supplementary Table 1 Food additives selected as firming agents and stabilizers (82–87).
