# Supplementary figures and images for: An Alum-Free Jellyfish Treatment for Food Applications
Source: Front Nutr. 2021 Aug 23;8:718798. doi: 10.3389/fnut.2021.718798 (PMC8419267; doi:10.3389/fnut.2021.718798)

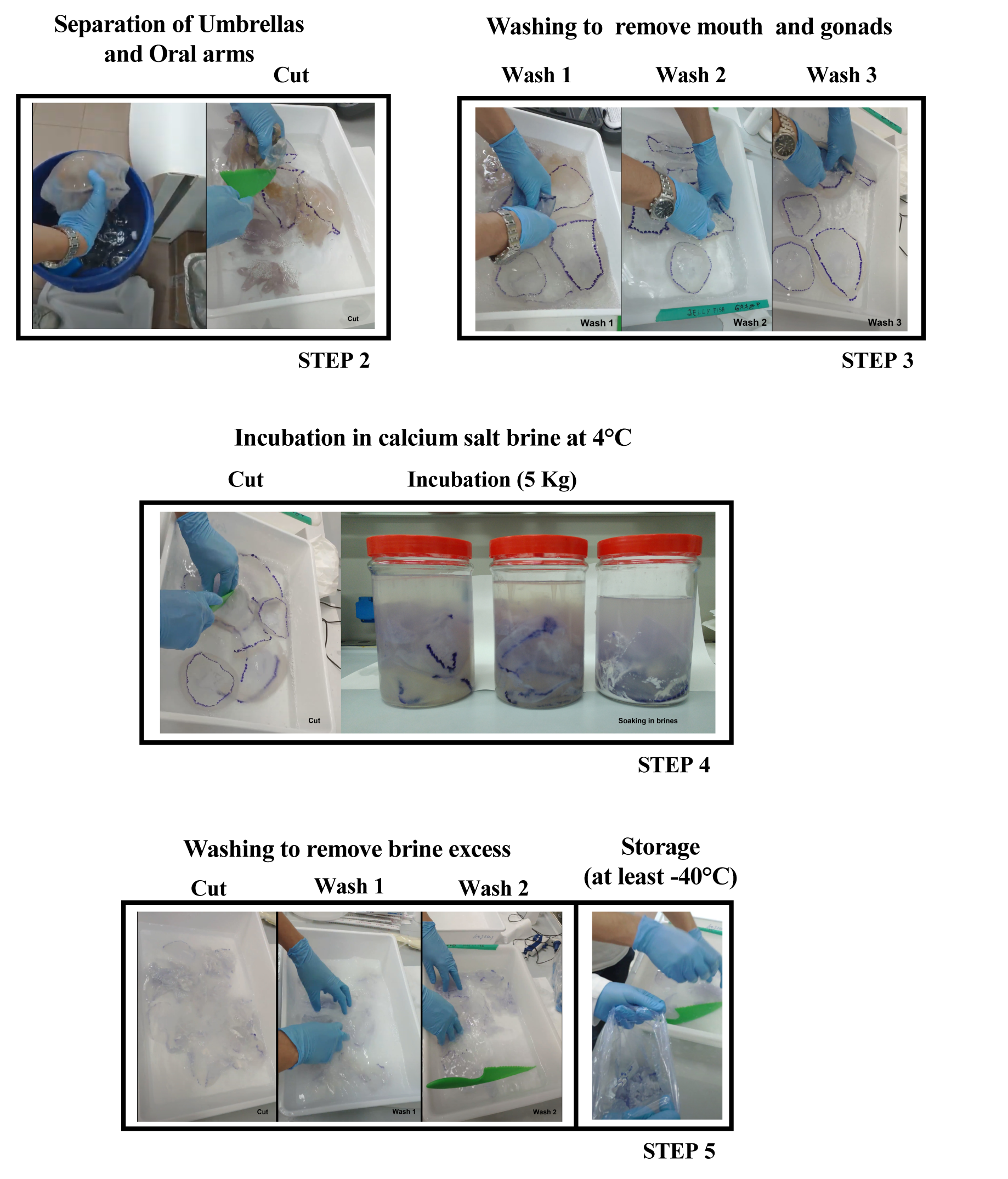

Supplement: Supplementary Figure 1 — Steps of JF processing (corresponding to steps 2 to 5 shown in Figure 4) to obtain semi-finished products for human food production. [file Image_1.TIF]
