# Supplementary material for: An Alum-Free Jellyfish Treatment for Food Applications
Source: Front Nutr. 2021 Aug 23;8:718798. doi: 10.3389/fnut.2021.718798 (PMC8419267; doi:10.3389/fnut.2021.718798)
Supplement: Supplementary Table 1 — Food additives selected as firming agents and stabilizers (82–87). [file Table_1.DOCX]

**Table S1**. Food additives selected as firming agents and stabilizers.

| **Product** | **International number** | **Classes** | **EU** | **U.S.A.** | **Australia and New Zealand** | **Applications** | **Approved** | | |
| --- | --- | --- | --- | --- | --- | --- | --- | --- | --- |
| **Calcium** |  |  |  |  |  |  | Halal | Kosher | Veg |
| Calcium acetate | E263 | Acidity regulator, Preservative, Stabiliser | 🗹 | 🗹 | 🗹 |  | Yes | Yes | Yes |
| Calcium carbonate | E170 | Acidity regulator, Anticaking agent, Stabiliser | 🗹 | 🗹 | 🗹 | Active ingredient in agricultural lime, usually the principal cause  of hard water | N/A | Yes | Yes |
| Calcium chloride | E509 | Firming agent, Stabiliser, Thickener | 🗹 | 🗹 | 🗹 | Firming agent used in canned vegetables, in firming soy bean curds into tofu and in producing  a caviar substitute from vegetable or fruit juices | Yes | Yes | Yes |
| Calcium citrate | E333 | Acidity regulator, Firming agent, Sequestrant, Stabiliser | 🗹 | 🗹 | 🗹 | Commonly used as a food additive (E333), usually as a preservative, but sometimes for flavor | Yes | Yes | Yes |
| Calcium gluconate | E578 | Acidity regulator, Firming agent, Sequestrant | 🗹 | 🗹 | 🗹 | Mineral supplement | Yes | Yes | Yes |
| Calcium lactate | E327 | Acidity regulator, Flour treatment agent | 🗹 | 🗹 | 🗹 | Used in foods (as a baking powder) and in medical treatment | Yes | Yes | Yes |
| Calcium lactylates | E482 | Emulsifier, Stabiliser | 🗹 | 🛈 | 🗹 |  | Maybe | N/A | N/A |
| Calcium lactobionate | 399 | Stabiliser | ⦸ | 🗹 | ⦸ | Food additive in cooking | N/A | Yes | Yes |
| Calcium phosphate | E341 | Acidity regulator, Antioxidant, Firming agent, Flour treatment agent, Foaming agent | 🗹 | 🗹 | 🗹 | Used in baking as a raising agent and in cheese products | Maybe | N/A | N/A |
| Calcium sulphate (E516) | E516 | Firming agent, Flour treatment agent, Sequestrant, Stabiliser | 🗹 | 🗹 | 🗹 | Common use in laboratory and industrial chemical. Used as a coagulant in products like tofu | N/A | Yes | Yes |
| Calcium sulfte | E226 | Antioxidant, Preservative | 🗹 | 🛈 | ⦸ |  | N/A | N/A | N/A |
| Calcium hydrogen sulphite (E227) | E227 | Antioxidant, Preservative | 🗹 | 🛈 | ⦸ | Acid salt with an acid behavior  in its aqueous solution | N/A | N/A | N/A |
|  | |  |  |  |  |  |  |  |  |
| Calcium tartrate | E354 | Acidity regulator | 🗹 | 🛈 | 🗹 | Byproduct of the wine industry, prepared from wine fermentation dregs | No | Yes | Yes |
| **Copper** |  |  |  |  |  |  |  |  |  |
| Cupric sulphate | 519 | Colour fixative, Preservative | ⦸ | 🛈 | 🗹 |  | N/A | N/A | Yes |
| **Iron** |  |  |  |  |  |  |  |  |  |
| Ferric ammonium citrate | 381 | Anticaking agent | ⦸ | 🗹 | 🗹 |  | Yes | N/A | Yes |
| **Magnesium** |  |  |  |  |  |  |  |  |  |
| Magnesium chloride | E511 | Firming agent, Stabiliser | 🗹 | 🗹 | 🗹 | Coagulant used in the preparation of tofu from soy milk | Maybe | Yes | Yes |
| Magnesium gluconate | 580 | Acidity regulator, Firming agent, Flavour enhancer | ⦸ | 🛈 | 🗹 |  | N/A | N/A | Yes |
| Magnesium sulphate | 518 | Flavour enhancer | ⦸ | 🗹 | 🗹 |  | Maybe | Yes | Yes |
| **Other** |  |  |  |  |  |  |  |  |  |
| Acacia gum (gum arabic) | E414 | Bulking agent, Carrier, Emulsifier, Glazing agent, Stabiliser, Thickener | 🗹 | 🗹 | 🗹 |  | Yes | Yes | Yes |
| Adipic acid | E355 | Acidity regulator | 🗹 | 🗹 | 🗹 | Food ingredient as a flavoring and gelling aid | N/A | Yes | Yes |
| Galaflow SPL 60-60/40 | | blend of sodium and potassium lactate at 60% and 40% concentration respectively. |  |  |  |  | Yes | Yes | Yes |
| **Ammonium** |  |  |  |  |  |  |  |  |  |
| Aluminium sulphate | E517 | Flour treatment agent, Stabiliser | 🗹 | 🗹 | ⦸ | Flocculating agent in the purification of drinking water and waste water treatment plants, and also in paper manufacturing | Maybe | Yes | Yes |
| Aluminium ammonium sulphate | E523 | Firming agent, Stabiliser | 🗹 | 🛈 | ⦸ | Used as acid source in baking powder for bakery products, baked at high temperature; colour stabiliser; used in industrial baking powder | Yes | Yes | Yes |
| Aluminium potassium sulphate | E522 | Acidity regulator, Stabiliser | 🗹 | 🛈 | ⦸ | Used as acid source in baking powder for bakery products, baked at high temperature; colour stabiliser; used in industrial baking powder | Yes | Yes | Maybe |
| Aluminium sodium sulphate | E521 | Firming agent | 🗹 | 🛈 | ⦸ | Form of alum used in the acidity regulator of food (E521) as well  as in the manufacture of baking powder | Yes | Yes | Yes |

⦸ Not permitted; 🛈 Not found; 🗹 Approved; N/A Not Applicable
